# Spatiotemporal regulation of angiogenesis/osteogenesis emulating natural bone healing cascade for vascularized bone formation

**DOI:** 10.1186/s12951-021-01173-z

**Published:** 2021-12-14

**Authors:** Xingzhi Zhou, Jiayu Chen, Hangxiang Sun, Fangqian Wang, Yikai Wang, Zengjie Zhang, Wangsiyuan Teng, Yuxiao Ye, Donghua Huang, Wei Zhang, Xianan Mo, An Liu, Peng Lin, Yan Wu, Huimin Tao, Xiaohua Yu, Zhaoming Ye

**Affiliations:** 1 Department of Orthopedic Surgery, The Second Affiliated Hospital, Zhejiang University School of Medicine, Hangzhou, Zhejiang 310000 People’s Republic of China; 2 Orthopedics Research Institute of Zhejiang University, Hangzhou, Zhejiang 310000 People’s Republic of China; 3 Key Laboratory of Motor System Disease Research and Precision Therapy of Zhejiang Province, Hangzhou, Zhejiang 310000 People’s Republic of China; 4 Department of Orthopedics, Renming Hospital of Wuhan University, Gaoxin 6th Road, Wuhan, Hubei 430000 People’s Republic of China; 5 School of Material Science and Engineering, University of New South Wales, Sydney, 2052 Australia

**Keywords:** Hydrogel, Mineral coating, Growth factor, Angiogenesis, Osteogenesis

## Abstract

Engineering approaches for growth factor delivery have been considerably advanced for tissue regeneration, yet most of them fail to provide a complex combination of signals emulating a natural healing cascade, which substantially limits their clinical successes. Herein, we aimed to emulate the natural bone healing cascades by coupling the processes of angiogenesis and osteogenesis with a hybrid dual growth factor delivery system to achieve vascularized bone formation. Basic fibroblast growth factor (bFGF) was loaded into methacrylate gelatin (GelMA) to mimic angiogenic signalling during the inflammation and soft callus phases of the bone healing process, while bone morphogenetic protein-2 (BMP-2) was bound onto mineral coated microparticles (MCM) to mimics osteogenic signalling in the hard callus and bone remodelling phases. An Initial high concentration of bFGF accompanied by a sustainable release of BMP-2 and inorganic ions was realized to orchestrate well-coupled osteogenic and angiogenic effects for bone regeneration. In vitro experiments indicated that the hybrid hydrogel markedly enhanced the formation of vasculature in human umbilical vein endothelial cells (HUVECs), as well as the osteogenic differentiation of mesenchymal stem cells (BMSCs). In vivo results confirmed the optimal osteogenic performance of our F/G-B/M hydrogel, which was primarily attributed to the FGF-induced vascularization. This research presents a facile and potent alternative for treating bone defects by emulating natural cascades of bone healing.

**Supplementary Information:**

The online version contains supplementary material available at 10.1186/s12951-021-01173-z.

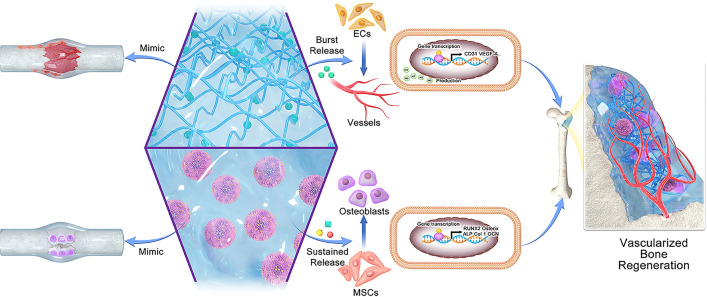

## Introduction

The treatment for bone defects resulting from malignancy resection or trauma is still a long-term clinical challenge, whereas bone tissue engineering has been emerging as one hopeful alternative to surmount this problem by using cell, scaffold, bioactive molecule, and biophysical enhancement [1–5]. As bone regeneration involves a series of complicated and well-orchestrated processes that involve the interaction and collaboration of multiple factors of bone marrow, bone matrix, and surrounding tissue, endogenous bone healing mechanism has been exploited to emulate natural healing cascade towards the bone formation [6]. Various growth factors (GFs) are present, each serving in a specified spatiotemporal pattern to orchestrate multiple biological processes during the four stages of bone healing, including inflammation stage, soft callus stage, hard callus stage, and bone remodelling stage. Thus, various growth factor delivery systems have been developed for providing biological cues to facilitate different aspects of bone healing [7]. For instance, bone morphogenetic proteins (BMPs) were delivered using a resorbable collagen sponge, while vascular endothelial growth factor (VEGF) was released via polymeric vehicles to enhance vascularization during bone healing [8, 9]. Thus, novel delivery systems that meet the needs of different stages of bone healing are urgently needed to optimize the outcomes of growth factor-based bone regeneration strategies.

Vascularization is of paramount importance to osteogenesis as the vascularity of the defective area is not only an essential process during bone repair, enabling the adequate supply of nutrients and mineralized components, but also functions in regulating cells as well as signaling molecules involved in osteogenesis [10, 11]. However, to achieve optimal bone formation, the coupling of osteogenesis and angiogenesis needs to be deliberately tailored in spatiotemporal manners [12]. To meet this demand, delivery systems with the ability to simultaneously supply osteogenic and angiogenic growth factors have been developed [13, 14]. Unfortunately, current delivery systems do not effectively imitate the pattern by which growth factors appear and act in vivo but act as depots for high concentrations of growth factors. Therefore, it remains a major challenge to couple osteogenesis and angiogenesis in bone tissue engineering. A variety of GF delivery vehicles are available using diverse polymers, in which growth factors can be immobilized by physical encapsulation, electrostatic interaction, and covalent attachment, etc. [15]. Depending on the incorporation method, the mechanism of growth factor release involves diffusion, dissolution, polymer erosion, or osmosis wetting phenomenon [16]. Advanced growth factor delivery systems able to co-deliver BMPs and angiogenic GFs, such as bFGF and VEGF, have been developed to achieve collaborative enhancements [17–19]. Unfortunately, due to the ineffectiveness in simulating the bone micro-environment, these delivery systems are still not the most optimal and thus discrepant compared to natural bone repair. Intriguingly, bone mineral composed of hydroxyapatite crystals has shown its capability to stabilize the protein and maintain its activity, even in hostile environments [20]. Inspired by this property of bone, prior studies have demonstrated that the growth of calcium phosphate mineral coatings on various template materials allowed the transport of multiple growth factors and diverse bioactive molecules [21–24]. The mineral coating preserves the growth factor's bioactivity and enables its controlled release. More importantly, with a similar composition and structure, the mineral coating closely resembles the bone microenvironment, thus mimicking the in vivo release of BMPs. Predictably, it is promising that the mineral coating emulates the natural bone healing cascade if combined with other delivery systems such as hydrogels.

**Scheme 1:**
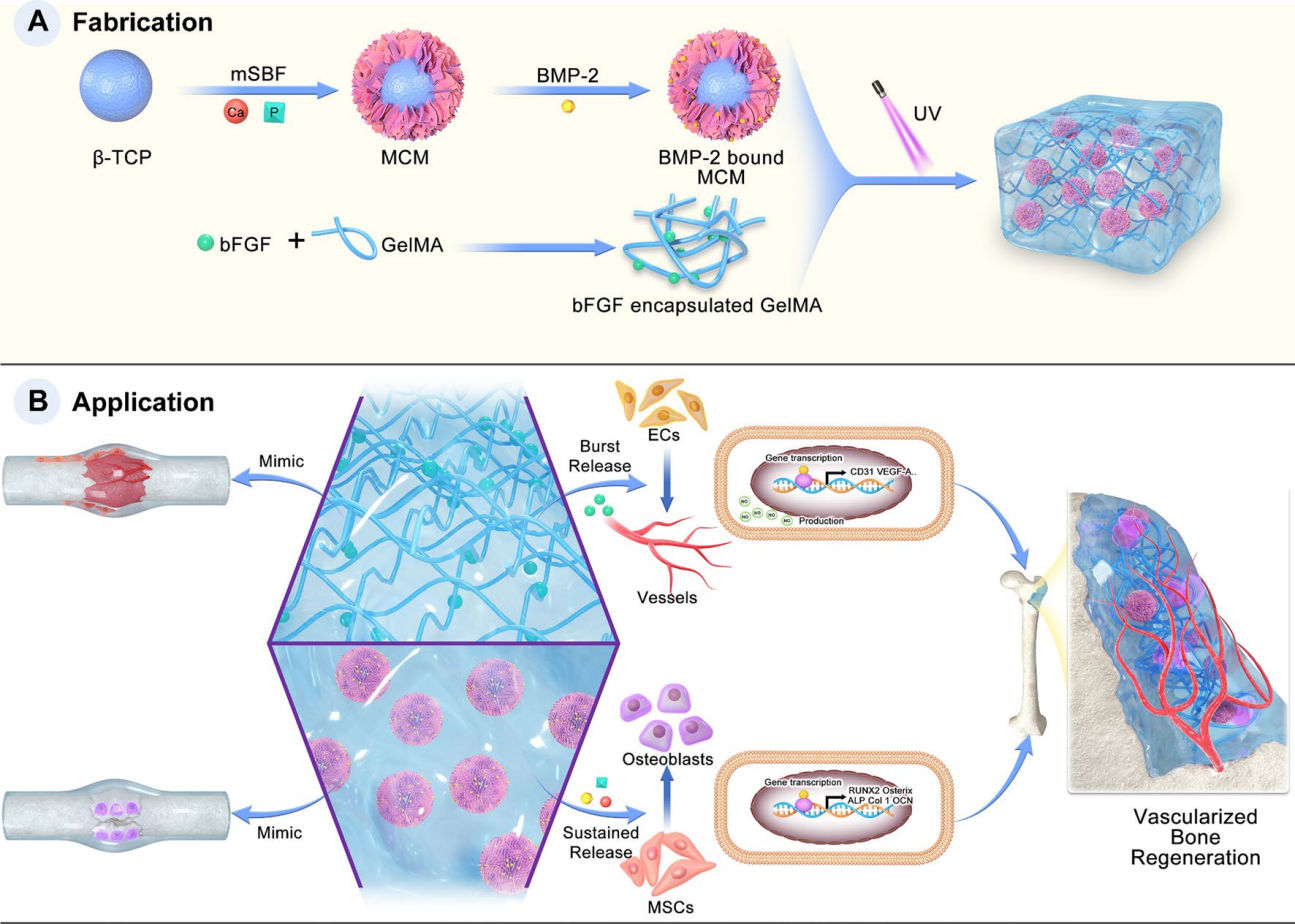
Schematic illustration of the fabrication and application of the F-G/B-M hybrid hydrogel

Based on the above rationale, we aimed to emulate the natural bone healing cascades by coupling the processes of angiogenesis and osteogenesis with a hybrid dual growth factor delivery system to achieve vascularized bone formation. In our study, GelMA hydrogel was used to incorporate BMP-2 bound MCM, and bFGF was physically encapsulated into the hydrogel network. In particular, the bFGF loaded in GelMA mimics growth factors from hematoma during the inflammation and soft callus phases of the bone healing process, while BMP-2 bound on MCM mimics growth factors in the hard callus and bone remodelling phases (Scheme 1). Thus, this hybrid hydrogel features a structure similar to natural bone and mimics the natural healing process to achieve the coupling of osteogenesis and angiogenesis and better-vascularized bone regeneration. In vitro experimental results demonstrated that a "burst" release of bFGF enhances the angiogenic process of HUVECs. In contrast, a more sustained release of BMP-2 promotes the process of osteogenesis in BMSCs. Follow-up in vivo studies indicated that our hybrid hydrogel had optimal bone regeneration performance, and specific experiments highlighted that this was achieved through enhanced angiogenesis. Therefore, we design this sophisticated strategy that emulates the natural healing cascades to enable vascularized bone regeneration, which has significant implications for the treatment of osseous defects.

## Materials and methods

### Fabrication and characterization of MCM

Mineral coated microparticle (MCM) was prepared based on a prior study [25]. β-TCP granules (particle size: 3–6 μm) were incubated in modified simulated body fluids (mSBF) for 7 days to prepare MCMs. The mSBF was obtained after adding the below reagents to distilled water heated to 37℃ in a certain sequence: 141 mM NaCl, 4.0 mM KCl, 0.5 mM MgSO_4_, 1.0 mM MgCl_2_, 4.2 mM NaHCO_3_, 20.0 mM HEPES, 5.0 mM CaCl_2_, and 2.0 mM KH_2_PO_4_. Subsequently, each 1 g of β-TCP granule was incubated in 500 mL of mSBF to create the mineral coating. The mSBF was renewed every day throughout the process to maintain a constant ion concentration for the growth of the mineral coating. The MCMs were flushed and lyophilized after incubation. The morphology of β-TCP and MCM was characterized by FE-SEM (Zeiss Sigma 300, Germany). The composition of the MCM was analyzed via EDX using the same SEM instrument. The phase composition of the MCM was determined by FTIR (Nicolet 10, Thermo Scientific, USA) and XRD (Bruker D8 Advance, Germany).

### Synthesis and characterization of GelMA

GelMA was prepared based on a prior study [26]. In short, 10.0 g gelatin (Sigma, USA) was slowly added into 100 mL of PBS (Gibco, USA) at 60 °C under constant stirring. Next, 8 ml of methacrylic anhydride in total was added slowly into the gelatin solution. After stirring for two hours at 60 °C, 100 ml of preheated PBS was poured into the above solution and stirred for 15 min. The supernatant was obtained by centrifugation and then dialyzed with a 12–14 kDa MWCO dialysis membrane (Spectrum Labs, USA) at 38 °C for 7 days. The freeze-dried GelMA was stored at − 20 °C. ^1^H NMR (Bruker 400 M, Germany) was used to identify the degree of methacryloyl substitution. The composition and morphology of the GelMA were examined by FE-SEM (Zeiss, Germany), FTIR spectroscopy (Bruker, Germany), and XRD (Thermo Scientific, USA).

### Preparation of BMP-2 bound MCM

To incorporate BMP-2 into MCMs, 2.5 mg MCMs were incubated in 1000 μL PBS solutions with recombinant human BMP-2 protein (rhBMP-2, R&D Systems, USA) for 4 h at 37 °C under continuous rotation. The BMP-2 loaded MCMs were subsequently centrifuged at 15,000 rpm for 3 min and rinsed twice. Following binding, the remaining amounts of BMP-2 were measured by microBCA protein assay (Beyotime, China). The binding efficiency was calculated from the change in BMP-2 concentration before and after binding. Calculating from the binding efficiency, the loading amounts of BMP-2 on MCM used for our study were 10 μg/sample according to some preliminary studies [26–28].

### Fabrication and characterization of hybrid hydrogel

To fabricate BMP-2-GelMA/bFGF-MCM hybrid hydrogel, the GelMA prepolymer was first obtained through a mixture of lyophilized GelMA macromolecules (5% w/v final) and photoinitiator LAP (EFL, China) (0.25% w/v) in PBS and heating at 65 °C until complete dissolution, which was subsequently filtrated via a 0.22 μm filter for sterilization. BMP-2 bound MCM and recombinant human bFGF protein (R&D Systems, USA) were subsequently mixed thoroughly with the above GelMA prepolymer solutions at the desired amount. The prepolymer solutions were exposed to ultraviolet light (30 mW/cm^2^, 365 nm) for 30 s to fabricate our hybrid hydrogel. GelMA/MCM hybrid hydrogels with or without growth factors (BMP-2 or bFGF) were fabricated similarly. Here, different groups of hybrid hydrogels were prepared for further experiments and named as the following description, **G** group: 5% (w/v) pristine GelMA, **G/M** group: 10% (w/w) MCM was mixed with 5% (w/v) GelMA, **F-G/M** group: 10% (w/w) MCM was mixed with 5% (w/v) GelMA containing 10 ng bFGF, **G/B-M** group: 10% (w/w) MCM containing 10 μg BMP-2 was mixed with 5% (w/v) GelMA, and **F-G/B-M** group: 10% (w/w) MCM containing 10 μg BMP-2 was mixed with 5% (w/v) GelMA containing 10 ng bFGF. To observe the morphology of hybrid hydrogel, the samples were examined in cross-section by FE-SEM (Zeiss, Germany). The structure and composition of the hydrogel were analyzed via FTIR (Bruker, Germany) and XRD (Thermo Scientific, USA).

Swelling and degradation.

Three G/M hydrogels were prepared using GelMA (5% w/v) with the MCM content ranging from 5.0 to 10.0 and 20.0 wt%, respectively, and they were denominated as GM-5, GM-10, and GM-20. Cylindrical hydrogel samples of 3 mm in height and 8 mm in diameter were prepared for the test.

The swelling ratio of different GelMA/MCM hydrogels was evaluated according to the methods previously reported [29]. All samples were submerged in PBS at 37 °C and then placed in a shaker at a speed of 200 rpm. Once reaching a predetermined time interval (1 h, 2 h, 4 h, 8 h, 12 h, and 24 h), The weight of the wet hydrogels was measured after removing the superficial water. The swelling ratio was determined from the formula: Swelling ratio (%) = (W_S_–W_i_)/W_i_, where W_i_ and W_s_ represented the original weight and the post-swelling weight, respectively.

Since MMP-8 (Type II collagenase) is responsible for in vivo degradation of GelMA after implantation [30]. To measure the degree of the in vitro degradation, we immersed the samples in PBS solution with collagenase II (20 U/μL) at 37 °C. The degradation rate was determined from the formula: Mass remaining (%) = (W_i_ – W_d_)/W_i_, where W_i_ and W_d_ represented the original weight of the hydrogels and the weight after freeze-drying at each predetermined time point, respectively.

### Mechanical characterizations

For compression and rheological tests, cylindrical hydrogel samples of 2 mm in height and 20 mm in diameter were prepared using the same method. The hydrogels were preconditioned in sterile de-ionized water for 24 h before testing. Compression experiments were conducted using a universal testing machine (CMT6103, MTS, USA) with a 1 mm/min loading speed. The strain amplitude sweep test (γ = 0.1–100%) was performed using a rheometer (Mars40, Thermo Scientific, USA) to detect the critical strain point that states the hydrogel is between fluid and solid.

### Release profiles of GFs and ions

To calculate the release profile of GFs and ions, samples were soaked in 1000 μL of Ca^2+^/Mg^2+^-free PBS under 120 rpm continuous vibration at 37 °C for over 4 weeks. The releasing buffer was collected at the desired time points. The amounts of bFGF and BMP-2 were quantified using a human bFGF quantikine ELISA kit (R&D Systems, USA) and a human BMP-2 quantikine ELISA kit (R&D Systems, USA), respectively, according to the manufacturer's instructions.

An Arsenazo III-based assay quantified the calcium amount released from the MCMs. In brief, 10 μL of releasing buffer was blended with 390 μL of Arsenazo III (0.4 mM) in Tris buffer (20 mM). The absorbance was then detected at 615 nm. The calcium concentrations were determined from a series of predetermined standards. The phosphate amount released from the MCMs was quantified by an acetone-acid-molybdate (AAM) based assay. In brief, 100 μL of releasing buffer was blended with an equivalent volume of AAM solution containing 10 mM ammonium molybdate, 5.0 N sulfuric acid, and acetone. The amount of phosphate was then measured by absorbance at 405 nm, and the phosphate concentrations were also defined by a series of predetermined standards.

### Cell culture

Cyagen Biosciences (Guangzhou, China) provided HUVECs and hBMSCs used for the experiment. HUVECs were cultured in DMEM (Gibco, USA) containing 10% fetal bovine serum (FBS, Gibco, USA) and 1% penicillin/streptomycin (P/S, Invitrogen, USA) at 37 °C with 5% CO_2_, while hBMSCs were cultured in α-MEM (Gibco, USA) containing 10% FBS and 1% P/S at 37 °C with 5% CO_2_. The medium was changed every two days, and the cells were passaged once reaching 80–90% confluence. Cells at passage 3 to 5 were used in all experiments.

### Cell viability

A Live/Dead assay kit (Beyotime, China) was used to assess cell viability. Briefly, different hydrogel samples (G and G/M) were sterilized and pretreated in the culture medium for 24 h. Next, HUVECs or hBMSCs (1 × 10^4^ cells/sample) were cultured on the sample surface for 1, 4 and 7 days. The inverted fluorescent microscope imaged the stained cells, and the number of cells was calculated by Image J software from 5 randomly chosen images.

### Cell proliferation and morphology

Cell proliferation was assessed by the CCK-8 assay kit (Dojindo, Japan). In brief, HUVECs or hBMSCs were seeded on the sample surface in densities of 2.5 × 10^3^ cells/sample for 1, 3, 5, and 7 days. At the defined point in time, each well was added with 200 μL of serum-free cell culture medium containing 10% CCK-8 and incubated for two hours. The absorbance of the solution was determined at 450 nm with a spectrophotometric microplate reader (Molecular Devices, USA). Further, cell adhesion and morphology were examined by staining the cytoskeletons with FITC-conjugated phalloidin (Invitrogen, USA) and the cell nuclei with DAPI (Beyotime, China). Briefly, cells were washed three times and fixed with 4% paraformaldehyde (PFA, Biosharp, China) for 30 min. Subsequently, after being permeabilized with Triton X-100, HUVECs and BMSCs were incubated with phalloidin (FITC) overnight and stained with DAPI for 5 min. Lastly, laser scanning confocal microscopy (LSCM, Leica, Germany) was employed to observe the labelled cells.

### Nitric oxide (NO) production

The NO production of HUVECs on different hydrogels was detected with the probe 3-amino-4-(aminomethyl)-2′,7′-difluorescein, diacetate (DAF-FM DA, Beyotime). The cells were cultured on different samples for 24 h, and the medium was replaced by 1 mL/well of the probe solution (5 μM). After incubation for 20 min in the dark, all cells on the samples were visualized using LSCM (Leica, Germany).

### Tube formation assay

For evaluating the capability of the released bFGF to promote angiogenesis, growth factor-reduced Matrigel (200 μL/well) (Corning, USA) was gelled in a tissue culture plate. HUVECs were cultured on the matrigel substrate for 5 × 10^4^ cells/well density, with the different samples (8 mm in diameter) immersed in the upper chamber of a 0.4 μm Transwell plate (Corning, USA). Additionally, a group of pure bFGF was assigned as a positive control group with the same amount of bFGF (100 ng). At the third or sixth hour, the cells were stained with Calcein AM and photographed by fluorescence microscope (Leica, Germany). The quantitative parameters were counted in five random fields using Image J software [31]

### Alkaline phosphatase (ALP) activity

To evaluate the osteogenic differentiation property induced by different hydrogels, hBMSCs were cultured at a density of 1 × 10^5^ cells/well in a 6-well Transwell plate (Corning, USA) while samples were placed in the upper chamber. After 48 h, the medium was changed to osteoinductive medium (OM) containing 10^−8^ M dexamethasone, 10 mM β-glycerol phosphate, and 50 μg/mL ascorbic acid (Sigma-Aldrich, USA). After incubation for 3 and 7 days, the cells were fixed and stained with BCIP/NBT working solution (Beyotime, China). The ALP activity was evaluated with the ALP Assay Kit (Beyotime, China). After co-incubation of the cell lysis substrate and p-nitrophenol at 37 °C for 30 min, the ALP activity was measured at 405 nm. Finally, the ALP levels were normalized to the total protein content measured by the BCA protein assay kit.

### Alizarin red staining

To highlight mineralized nodes, Alizarin Red staining (ARS) was employed. hBMSCs were cultured in OM as described above. After culturing for 14 and 21 days, the BMSCs were fixed and rinsed three times with distilled water. The cells were further incubated with ARS Staining Solution (Beyotime, China) for 30 min. Stained cells were observed by an inverted light microscope (Olympus, Japan). To analyze quantitatively, the mineralization was dissolved with 10% cetylpyridinium chloride (Sigma, USA), and the absorbance of the lysate was subsequently recorded at 562 nm.

### BMSCs immunofluorescence

After two days of incubation in culture medium, followed by three days of incubation in osteoinductive medium, hBMSCs were rinsed and then fixed for 15 min. The cells were permeabilized for 20 min and blocked with 10% goat serum (Invitrogen, US) for 1 h at 37 ℃. After being rinsed three times, the cells were probed with the primary antibodies against RUNX2 (Cell Signal Technology, USA) overnight at 4 ℃ and then incubated with phalloidin solution (1:1000) and an Alexa Fluor-coupled secondary antibody (Beyotime, China, 1:400) for 2 h. Lastly, the nucleus was counterstained with DAPI for 3 min, and the cells were imaged by the fluorescence microscope.

### Quantitative real-time PCR analysis

For osteogenic evaluation, hBMSCs were seeded on the G, G/M, G/B-M, F-G/B-M hydrogel at a 1 × 10^5^ cells/sample density. BMSCs in pure culture medium was regarded as the blank control group. Following 3, 7 and 14 days of culture, total RNA was extracted by RNAiso plus (Takara, Japan). Complementary DNA (cDNA) was synthesized from 1 μg RNA by HiScript III All-in-one RT SuperMix (Vazyme, China). RT-qPCR was performed by SYBR Green Real-Time PCR Master Mixes (Thermo, USA). Two-step cycling conditions were as follows: 95 °C for 30 s, followed by 40 cycles at 95 °C for 5 s and 60 °C for 30 s.

For angiogenic evaluation, HUVECs in the pure medium was regarded as the blank control group, and the cells were seeded on the G, G/M, F-G/M, F-G/B-M hydrogel at a 5 × 10^4^ cells/sample density. After three days, the expression of angiogenic genes was assessed with a procedure similar to that described above. β-actin was selected as an internal control, and the primer sequences used were described in Additional file 1: Table S1.

### Rat calvarial critical-size defect model and hydrogel implantation

A rat calvarial critical-size defect model was performed in order to explore the osteogenic ability in vivo [32]. The animal care and surgical procedures were carried out following protocols approved by the Ethics Committee of the Second Affiliated Hospital of Zhejiang University. Male Sprague–Dawley (SD) rats were obtained from SLAC Laboratory Animal Co. Ltd (Shanghai, China). After adaptation over 1 week, rats with a weight of 280–300 g were chosen for the experiment. After anesthesia, the skin was sterilized, and a vertical incision was established on the skull. Then, two bilateral defects (5 mm in diameter) were created with a dental trephine drill. The calvarial defects were either covered with different hydrogels or left untreated. The holes were flushed after removing the bone, and the hydrogels were randomly positioned into the defects. A total of thirty-six animals were randomly divided into the following six groups: (1) empty defect (Sham), (2) G, (3) G/M, (4) F-G/M, (5) G/B-M, (6) F-G/B-M.

### Micro-CT analysis

After operation for 4 and 8 weeks, the rats were dosed intraperitoneally with 4% pentobarbital and euthanized. The specimens were collected and fixed in 10% formalin for the subsequent analysis. First, Micro-CT (Skyscan 1172, Bruker, USA) was applied to the analysis of the three-dimensional structure of regenerated bone. Reconstruction of 3D images for the calvarium was performed via the affiliated system software. The bone tissue volume/total tissue volume (BV/TV), bone mineral density (BMD), trabecular thickness (Tb. Th), and trabecular separation/spacing (Tb. Sp) were calculated and analyzed.

### Bone histology and immunohistochemistry

For histological analysis, the calvarial specimen was fixed with 4% neutral PFA for 2 days and subsequently decalcified by 10% EDTA with a solution change twice weekly for four weeks. The samples were dehydrated through ethanol and xylene, embedded in paraffin and sectioned into 5 μm sections. The bone tissue sections were stained with hematoxylin and eosin (HE) and Masson's trichrome (MT) staining and observed by an inverted optical microscope (Leica, Germany).

For immunostaining, bone sections were permeabilized for 20 min and blocked for 30 min. Then, the tissue sections were probed with the primary antibodies overnight at 4 °C. Subsequently, the sections were incubated with an appropriate Alexa Fluor-coupled secondary antibody (Molecular Probes, USA, 1:400) for 2 h. Nuclei were also counterstained with DAPI for 3 min. Lastly, the sections were photographed by the fluorescence microscope (Leica, Germany).

### Statistical analysis

All experiments were performed in triplets unless otherwise indicated. All data were expressed as the mean ± standard deviation (SD). Statistical differences were analyzed using a one-way analysis of variance (ANOVA) followed by Tukey's multiple comparisons test. The significant difference was set at p < 0.05.

## Result

### Fabrication and characterization of MCM, GelMA, and hybrid hydrogel

**Fig. 1:**
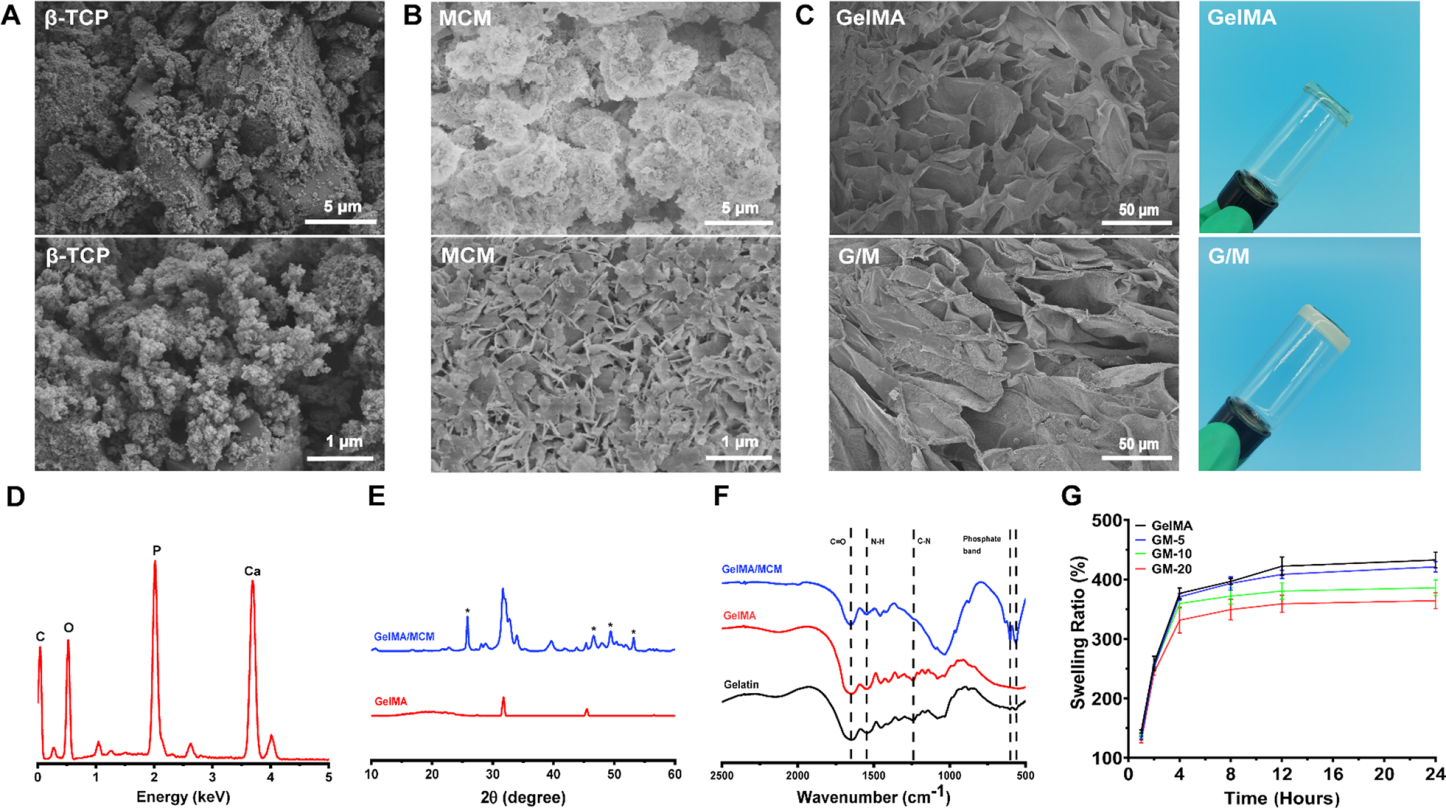
Morphology and characterizations of MCM, GelMA, and GelMA/MCM hydrogel. SEM micrographs of **A** β-TCP and **B** MCM. **C** SEM micrographs and digital photographs of GelMA and GelMA/MCM. **D** EDS of MCM. **E** XRD and **F** FTIR spectra of GelMA and GelMA/MCM. **G** Swelling test of GelMA and GelMA/MCM

To emulate bone healing cascade via an engineering approach, we constructed a multifunctional growth factor delivery system able to present specific growth factors in a spatiotemporal way leveraging the controlled release properties of both hydrogels and mineral coatings as shown in Scheme 1. Firstly, mineral-coated microparticles (MCMs) were first prepared by incubating β-TCP granules in mSBF, as reported in a previous study [33]. As illustrated in Fig. 1A and B, the MCM was homogeneously covered with a mineral coating featuring a typical plate-like nanostructure that mimics certain structural characteristics of natural bone minerals. The components of the mineral coatings were identified as carbonate-substituted hydroxyapatite (cHAP) via FTIR and XRD (Additional file 1: Fig. S1A, B). EDX further showed that the Ca/P ratio for the mineral coating was 1.88, indicating a certain extent of carbonate substitution occurred (Fig. 1D). ^1^H NMR and FTIR confirmed the successful chemical modification of gelatin: chemical groups between 5.0 to 6.0 ppm typical of methacrylates were observed in ^1^H NMR spectrum (Additional file 1: Fig. S1C), while obvious absorption peaks of C–N (1241 cm^−1^), N–H (1560 cm^−1^), and C=O (1654 cm^−1^) were observed in FTIR spectrum (Fig. 1F). MCMs were then added into GelMA prepolymer solution and photo-crosslinking under UV to form a hybrid hydrogel intended for growth factor loading and release

Figure 1C showed the digital photograph and SEM photograph of GelMA/MCM hybrid hydrogel. After mixing with MCMs, the appearance of GelMA hydrogel changed from translucent to opaque and opalescent. SEM results show that GelMA hydrogel and GelMA/MCM hydrogel features sponge-like macroporous structures and connected channels with MCM particles visible in the latter. XRD analysis and FTIR spectrum further confirmed the presence of MCMs in the GelMA network. As shown in Fig. 1E, F, compared with the XRD result and FTIR spectra of GelMA**,** characteristic peaks associated with carbonate and phosphate could be identified in GelMA/MCM.

The swelling ratio reflects the water absorbency and structural stability of the hydrogel. As shown in Fig. 1G, after a 24 h swelling experiment, results suggested a slight decrease in the swelling ratio after the addition of MCM. Still, all tested hydrogels maintained outstanding water retention capability. We then used weight loss testing for further exploration of the structural stability. The mass loss rate of the GelMA/MCM hydrogels with various MCM proportions was slightly higher than the mass loss rate of GelMA hydrogel, and the mass loss rate increased with the rising proportion of MCMs (Additional file 1: Fig. S1D), suggesting the excellent biodegradability of this hybrid hydrogel. In addition, the degradation of GelMA lasted for over four weeks, which matched the formation of bone. Finally, are shown in Additional file 1: Fig. S1E, we evaluated the mechanical behaviours of samples. The compressive modulus of GelMA/MCM hydrogels was significantly greater than that of GelMA at the same GelMA content, indicating that the addition of MCMs enhanced the mechanical property of GelMA. Additional file 1: Fig. S1F showed the strain amplitude sweep test result. The loss modulus curve approached the storage modulus curve, suggesting the hydrogels were in a state between solid and liquid near the critical point.

### Biocompatibility of hydrogel in vitro

**Fig. 2:**
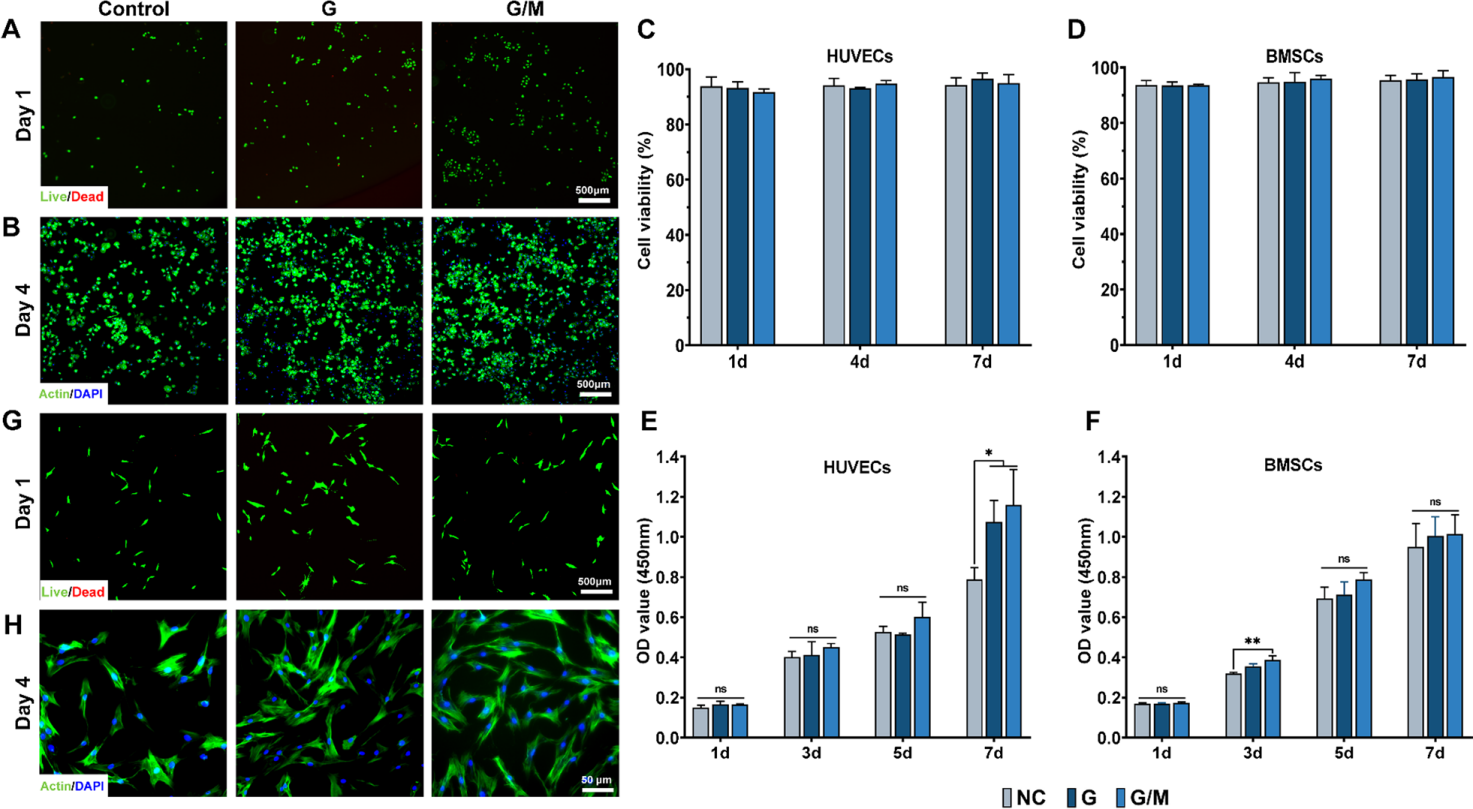
Biocompatibility evaluation of GelMA/MCM hydrogel. Representative Live/Dead images of **A** HUVECs and **G** BMSCs at day 1. Quantitative analysis of cell viability of **C** HUVECs and **D** BMSCs. Representative F-actin/DAPI images of **B** HUVECs and (H) BMSCs at day 4. Cell counting kit-8 assay of **E** HUVECs and **F** BMSCs at day 1, 3, 5, and 7. Statistically significant differences are indicated with *p < 0.05, **p < 0.01

HUVECs and hBMSCs were used to evaluate the biocompatibility of the hydrogels as they are actively involved in bone healing. Live/dead staining suggested that the majority of the seeded cells were still viable, and few dead cells were detected. After culturing HUVEC and BMSC on the samples for 1, 4, and 7 days, the staining indicating either GelMA or GelMA/MCM hydrogels exhibited favourable cell viability (Fig. 2A, G; Additional file 1: Fig. S2). The results of the quantitative analysis showed no significant differences between the blank and treated groups (P > 0.05), and all hydrogels provided good support for the survival (> 90% cell viability) of both cell types (Fig. 2C, D). For quantifying the proliferation of both types of cells among different groups, a CCK-8 assay was performed on different days. For HUVECs, no statistical differences in OD values were found between the groups until day 7 (Fig. 2E). For hBMSCs, statistical differences were observed on day 3 (Fig. 2F). Since GelMA retains the RGD sequences that facilitate cell adhesion and spreading, we also performed cytoskeleton staining to examine the extension and morphology of the cells. As shown in Fig. 2B and H, HUVECs and hBMSCs cultured on the hydrogel both displayed well-stretch morphology. All these results suggest that the hydrogels used had negligible cytotoxicity and demonstrated great biocompatibility.

### In vitro release behaviour

**Fig. 3:**
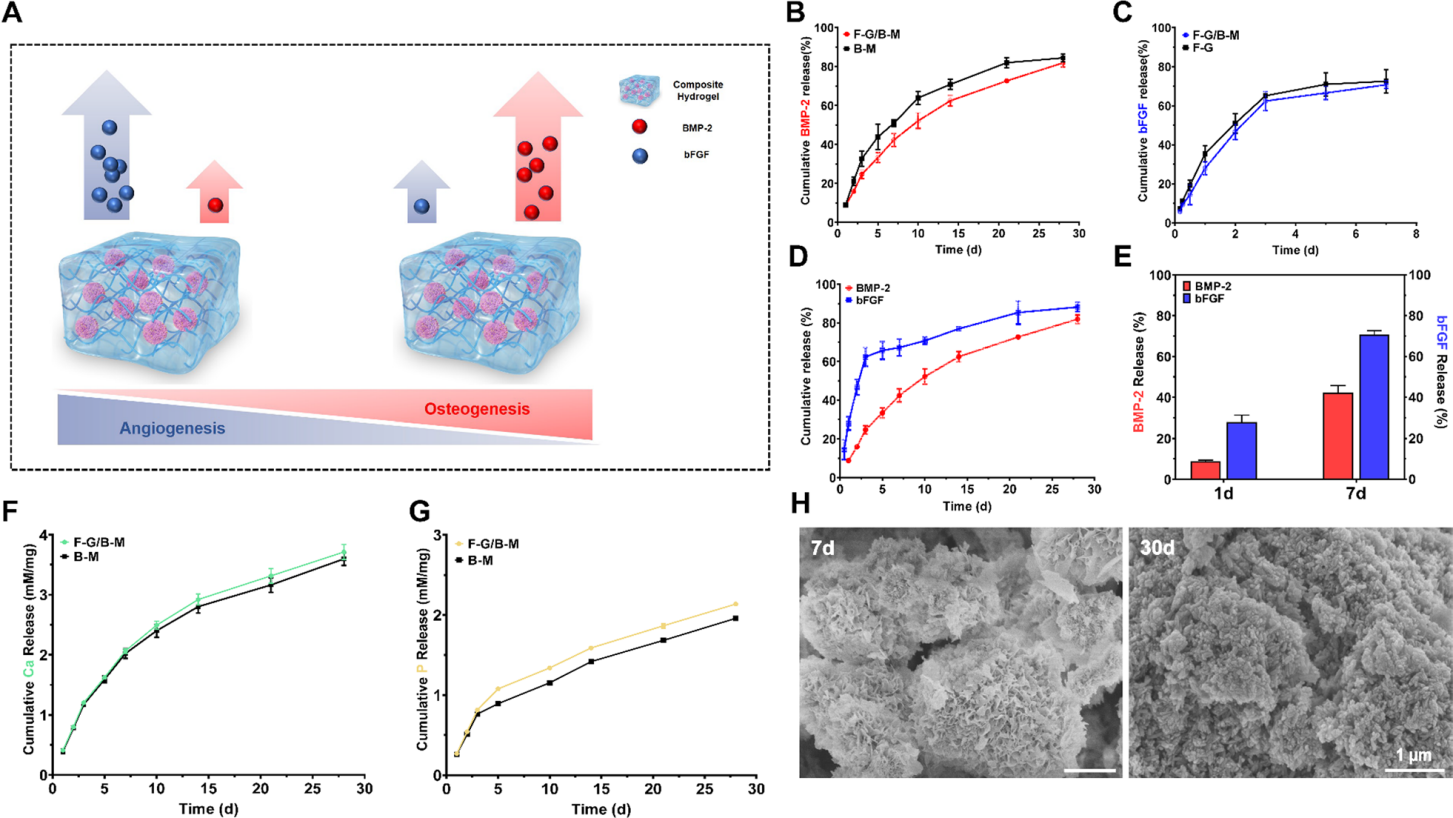
**A** Schematic illustration of the GFs release behavior. **B** Release kinetics of BMP-2. **C** Release kinetics of bFGF. **D**, **E** Release kinetics of BMP-2 and bFGF. **F** Release kinetics of calcium ion. **G** Release kinetics of phosphate ion. **H** SEM micrographs of MCM after incubation in PBS for 7 days and 30 days

To achieve differentiated release behaviours of different growth factors, BMP-2 was bound on MCMs to mimic its release kinetics from bone matrix, while bFGF was embedded in GelMA matrix to mimic its diffusion pattern from hematoma or surrounding soft tissue. As shown in Fig. 3A, BMP-2 was bound to MCM surface via electrostatic interactions with binding efficiency of 63.3%, then BMP-2 bound MCMs were mixed with GelMA prepolymer solution loaded with bFGF to achieve BMP-2/bFGF dual release system with different release kinetics. As depicted in Fig. 3B–E, a different release profile of the two GFs was observed. BMP-2 exhibited a sustained release without "burst" release. (Fig. 3B, D). Moreover, there was no significant hysteresis effect of mixing with GelMA on the release of GFs. On the other hand, bFGF displayed a two-stage release profile with a high initial rapid release within the first three days and relatively slow release over the next four weeks (Fig. 3C, D). In addition, the release of bFGF was not affected obviously with the addition of MCM. Specifically, within one day, the release percentages for BMP-2 and bFGF were 28.0 ± 3.4% and 8.8 ± 0.6%, respectively. By day 7, the release percentages were 42.4 ± 3.4% and 67.2 ± 4.3%, respectively (Fig. 3E). The degradation of mineral coatings is closely tied to the release of BMP-2, which was then characterized in terms of the release of phosphate and calcium. It was observed that the release of calcium and phosphate exhibited a sustained release process as well (Fig. 3F, G), which matched the release profile of BMP-2. SEM photograph further illustrated the degradation of the mineral coating. On day 7 during the BMP-2 release process, the plate-like nanostructure of the coating appeared a certain degree of disruption, while by day 30, the structure almost vanished and the substrate was exposed (Fig. 3H), suggesting the degradation of the mineral coating over time may be used to control growth factor release.

### In vitro osteogenic evaluation of hBMSCs

**Fig. 4:**
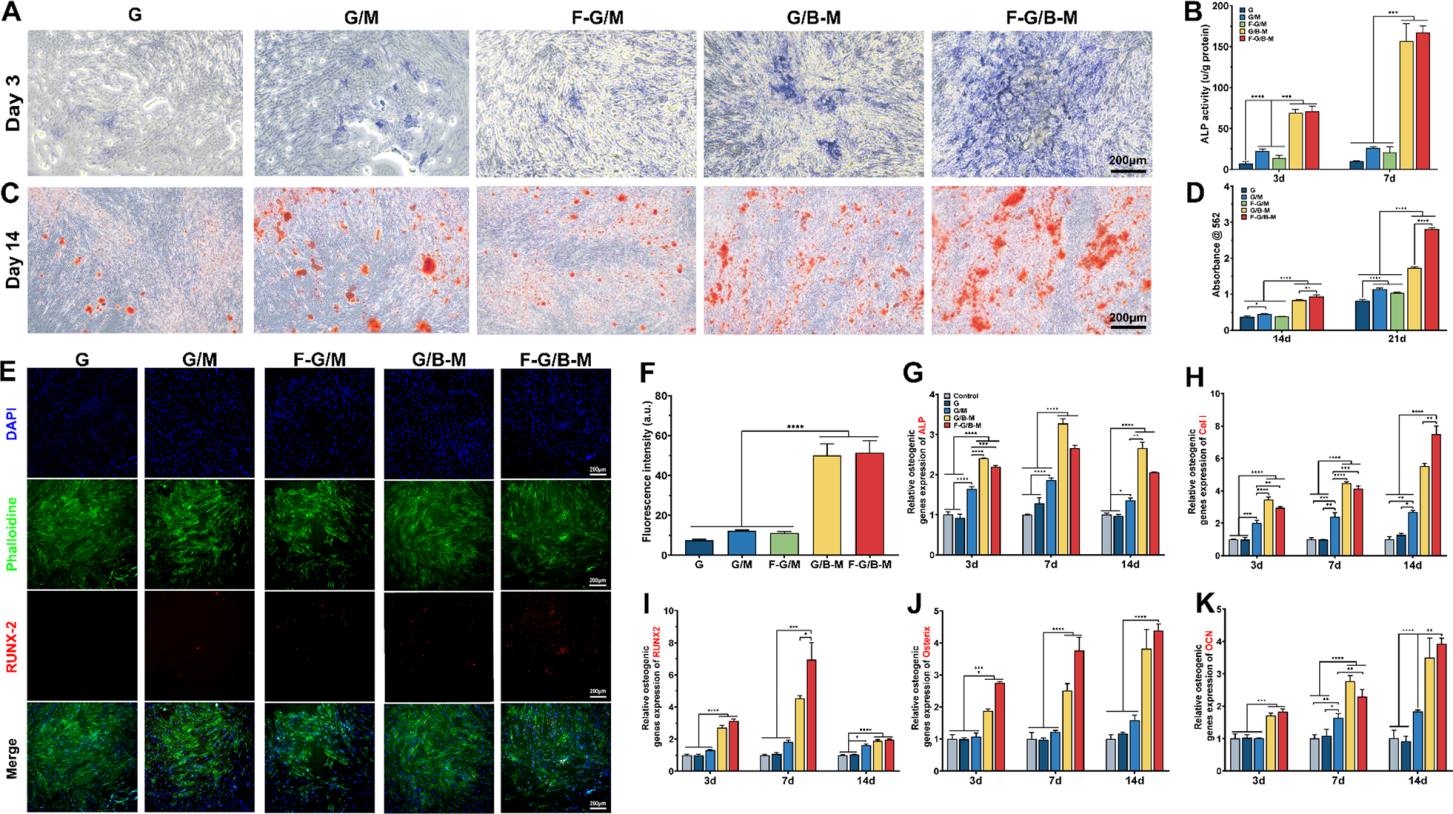
In vitro osteogenesis potential. **A** Representative ALP staining images on day 3. **B** Quantitative analysis of ALP activity of BMSCs. **C** Representative ARS images on day 14. **D** Quantitative analysis of calcium deposition of BMSCs. **E**, **F** Osteogenesis of BMSCs was measured by immunofluorescence assays for Runx2 expression on day 4, with the nucleus stained in blue and OCN stained in red. qRT-PCR analysis of osteogenesis-related gene expressions including **G** ALP, **H** Col-1, **I** Runx2. **J** Osterix and **K** OCN. Statistically significant differences are indicated with *p < 0.05, **p < 0.01, ***p < 0.001, ****p < 0.0001

To assess the impact of GFs releasing hydrogels on BMSCs osteogenic differentiation in vitro, we performed ALP staining, ARS, immunofluorescence staining of RUNX2, and quantitative real-time PCR analysis after culturing cells with hydrogels in Transwell chambers. As depicted in Fig. 4A and Additional file 1: Fig. S3, after incubation for 3 and 7 days, the staining intensity was significantly stronger in the F-G/B-M and G/B-M groups, indicating that the BMP-2 containing groups had a more significant expression of ALP. Quantitative analysis of ALP activity further confirmed that cells in the G group demonstrated a significantly lower ALP activity over the incubation period. The groups with BMP-2 exhibited substantially higher ALP activity but most obvious in the F-G/B-M group, which may be related to a combined effect of the dual GFs. As reported by literature, bFGF effectively stimulated cell proliferation of BMSCs, which may combine with BMP-2 to boost ALP production at the early phase of osteogenic differentiation [34]. Additionally, the groups containing MCM but not BMP-2 (G/M and F/G-M) exhibited higher ALP activity than the G group, presumably resulting from the release of calcium and phosphate. A similar tendency was seen in the ARS at day 14 and 21, a marker of calcium deposition relevant to later stage osteogenesis (Fig. 4C, Additional file 1: Fig. S3). The F-G/B-M group showed the most mineralized nodule formation, while the mineralized nodules with irregular shapes were joined into one piece. The G/M and F/G-M group also displayed more mineralized nodules with heterogeneous red colour compared to the G group. Quantification of the dissolved mineralized matrix demonstrated a consistent result (Fig. 4D). The mineralization activity was significantly higher in the F-G/B-M and G/B-M groups.

To investigate the effects of hybrid hydrogels on the expression of genes and proteins relevant to osteogenic differentiation, we first assessed the expression of RUNX2 after three days of culture by immunofluorescence staining. As shown in Fig. 4E, obvious red fluorescence was observed in the F-G/B-M and G/B-M groups. Quantitative analysis of fluorescence intensity further suggested that the F-G/B-M and G/B-M groups showed significant statistical differences in the expression of RUNX2 compared to the other three groups (Fig. 4F). Figure 4G–K displayed the gene expression of ALP, RUNX2, Col-1, osterix, and OCN of hBMSCs on different hydrogels after 3, 7 and 14 days of incubation. Notably, the hydrogel with BMP-2 exhibited significant up-regulation of these osteogenic gene expressions.

### In vitro angiogenic evaluation of HUVECs

**Fig. 5:**
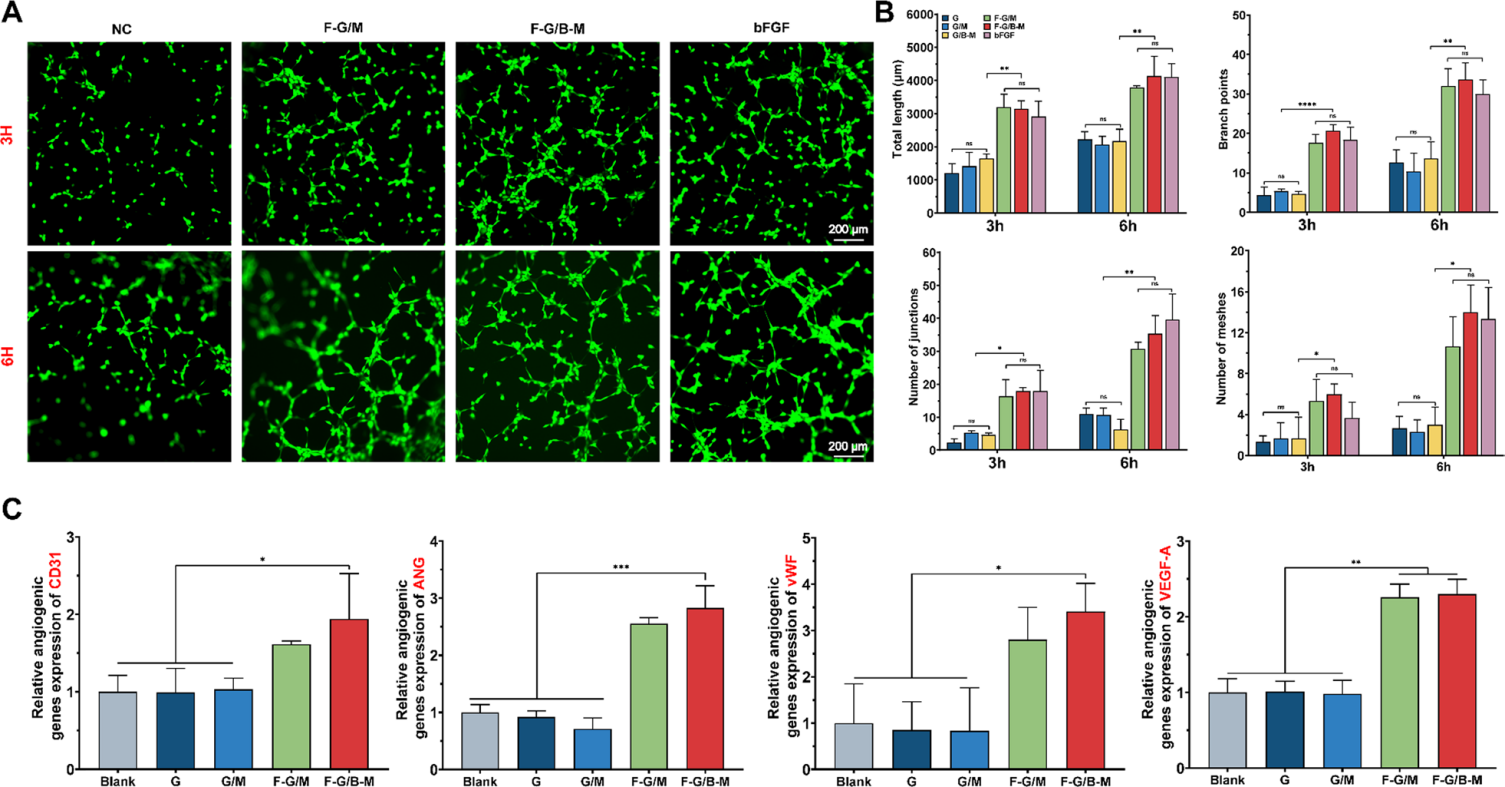
In vitro angiogenesis potential. **A** Representative fluorescence images of HUVECs after coculture for 3 and 6 h. **B** Quantification of total length, branch points, number of junctions, and number of meshes in HUVECs. **C** qRT-PCR analysis of angiogenesis-related gene expressions including CD31, ANG, vWF, and VEGF-A. Statistically significant differences are indicated with *p < 0.05, **p < 0.01, ***p < 0.001, ****p < 0.0001

A tubular structure formed by endothelial cells (ECs) is a vital process for the regeneration of functional vasculature [35]. Consequently, we conducted tube formation experiments for evaluating the influence of hybrid hydrogels on the angiogenesis process of HUVECs with the bolus bFGF group as a positive control (Fig. 5A). After culturing for 3 h, several distinct and vascular-like structures were observed in the F-G/M and F-G/B-M group, which were rarely seen in negative control groups (G, G/M, and G/B-M group). At the sixth hour, small tubes combined into large ones, and more tubules were induced with the presence of bFGF. Meanwhile, only a few inapparent tubes were identified in negative control groups. Total length, branch points, number of junctions, and number of meshes were further calculated from images. As shown in Fig. 5B, the total tube length of the F-G/B-M group was 3145 ± 243.0 μm at 3 h and 4139 ± 590.4 μm at 6 h, a significant increase compared to the G group (1212 ± 281.0 μm at 3 h and 2226 ± 231.9 μm at 6 h). The results of the positive control group (2912 ± 460.1 μm at 3 h and 4110 ± 396.6 μm at 6 h) were the same as that of the F-G/B-M group. The tendency of branch points, number of junctions, and number of meshes were generally consistent with that of total length, and they all increased with culturing time, suggesting its superior capacity to induce angiogenesis. Besides, a similar trend was found for the expression of angiogenesis-related genes, including cluster of differentiation 31 (CD31), angiogenin (ANG), von Willebrand Factor (vWF), and vascular endothelial growth factor A (VEGF-A) (Fig. 5C).

The intracellular nitric oxide (NO) expression level was assessed as an indicator of angiogenic function in endothelial cells [28]. As demonstrated in Additional file 1: Fig. S4, a high level of intracellular NO expression was found in the F-G/M and F-G/B-M group, suggesting that the initially high levels of bFGF play an important role in rapid vascularization.

### In vivo bone regeneration performance

**Fig. 6:**
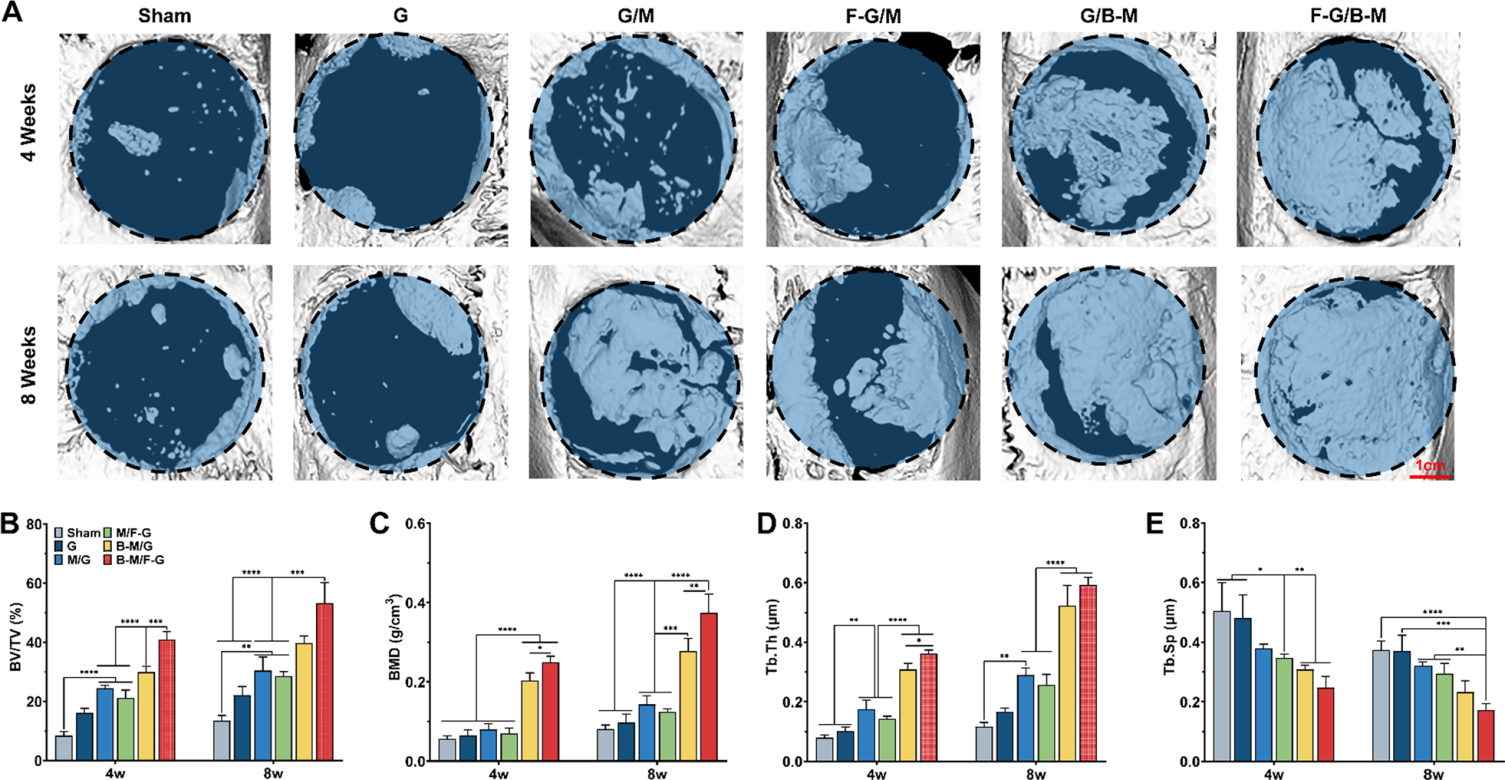
In vivo bone regeneration therapeutic efficacy. **A** 3D reconstructed micro CT images of the new bone formation after implantation for 4 and 8 weeks. Quantitative statistic of **B** BV/TV, **C** BMD, **D** Tb.Th, and **E** Tb.Sp of the newly formed bone at week 4 and week 8. BV/TV: bone volume fraction; BMD: bone mineral density; Tb.Th: trabecular thickness; Tb.Sp: trabecular separation. Statistically significant differences are indicated with *p < 0.05, **p < 0.01, ***p < 0.001, ****p < 0.0001

Following encouraging results of in vitro experiments, we conducted further in vivo studies of bone regeneration. The defects were left vacant with no treatment in the sham group. Micro-CT was conducted at 4 and 8 weeks postoperatively for analyzing the bone healing effect from macro aspects. As illustrated in three-dimensional reconstruction images (Fig. 6A), the new bone in the defective area was presented and grew from the peripheral part to the central. By 4 weeks, there was only very little obscured bone surrounding the defect in the sham and G groups, and a few new bone growths in the G/M group as well as F-G/M group, while more than half of the lesion was covered by new bone in the G/B-M as well as the F-G/B-M group. In the eighth week, only a few new bone coverage areas remained in the sham and G group, while the bone content was considerably higher for the F-G/B-M group than for the other groups. Micro-architectural parameters were used to quantify new bone formation. Figure 6B–E showed that BV/TV and BMD values were greatest in the F-G/B-M group. In the analysis of BV/TV, the values of the F-G/B-M group (40.9 ± 1.54% at 4 weeks and 53.2 ± 7.03% at 8 weeks) were more than 4 times those of the sham group (8.40 ± 0.78% at 4 weeks and 13.43 ± 1.80% at 8 weeks), and the values of the G/M group were (24.5 ± 9.0% at 4 weeks and 30.4 ± 4.54% at 8 weeks) were almost three times that of the sham group. Eight weeks post-operation, defects treated with F-G/B-M hydrogel also exhibited higher (0.59 ± 0.02 μm) Tb. Th and lower (0.17 ± 0.02 μm) Tb. Sp than those treated with sham surgery or other hybrid hydrogels.

**Fig. 7:**
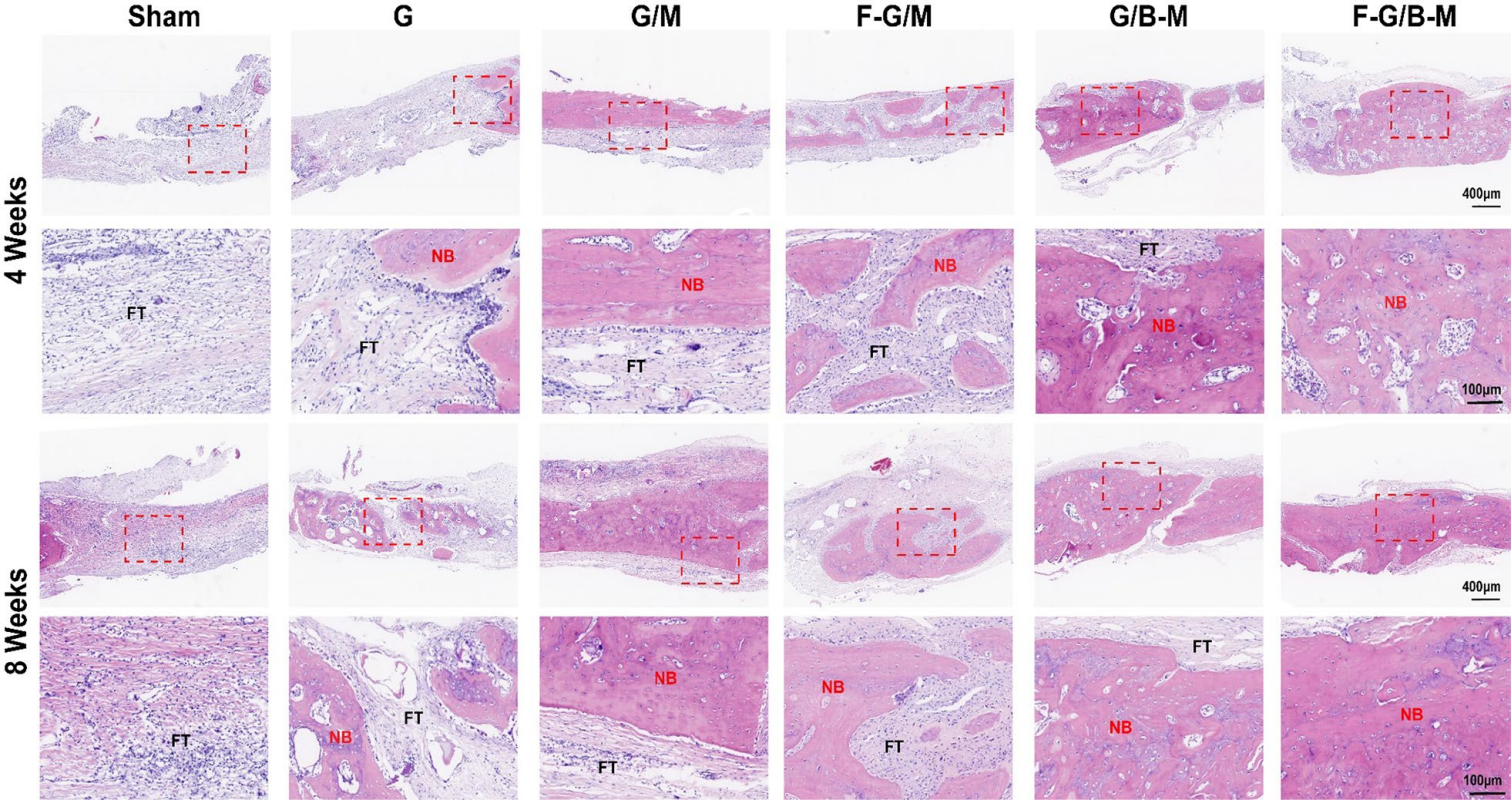
Hematoxylin and Eosin (H&E) staining of newly formed bone in the defect after implantation with different hydrogels for 4 and 8 weeks. Representative images were observed by low magnification and high magnification, showing the newly formed tissue, including the fibrous tissue (FT) and newly mineralized bone tissue (NB)

**Fig. 8:**
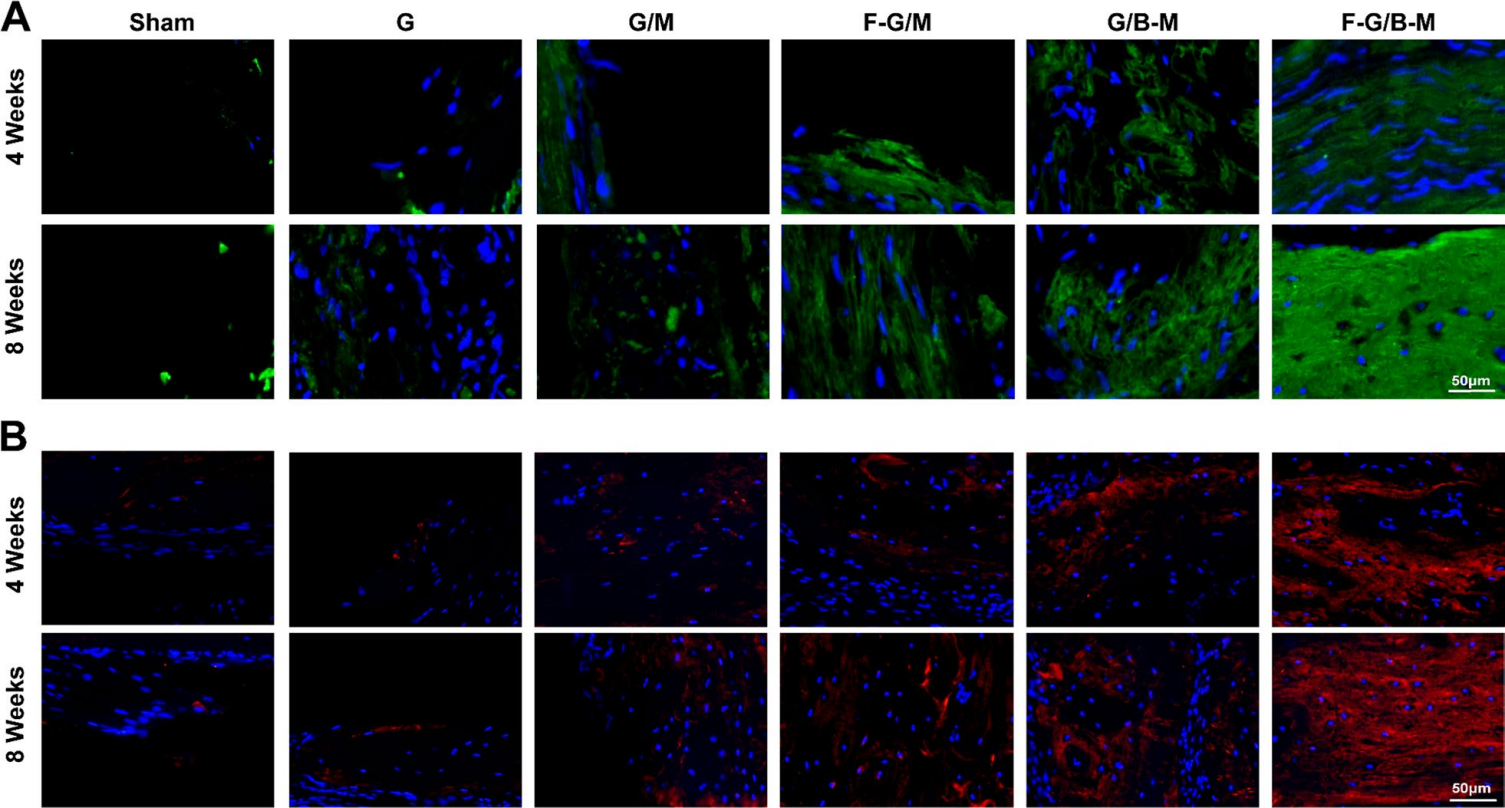
Evaluation of in vivo vascularized osteogenesis at week 4 and 8. **A** Representative immunofluorescence staining images of Col-1 (green) and nuclei (blue). **B** Representative immunofluorescence histochemical staining of OCN (red) and nuclei (blue)

**Fig. 9:**
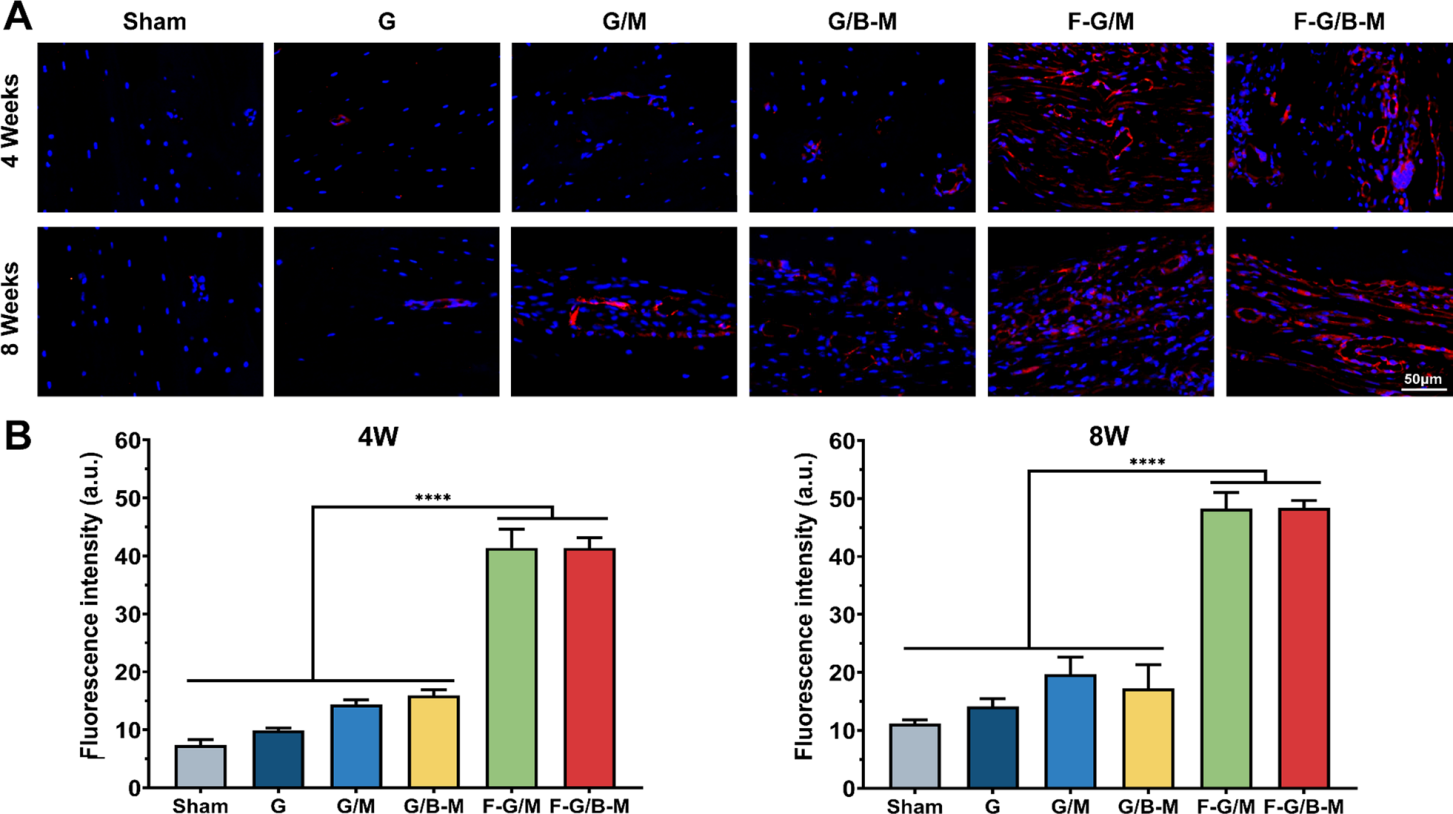
Evaluation of in vivo vascularized osteogenesis at week 4 and 8. **A** Representative immunofluorescence staining mages of bone vessels stained for CD31(red) and nuclei (blue) at week 4 and 8. **B** The semi-quantitative results of CD-31. Statistically significant differences are indicated with *p < 0.05, **p < 0.01, ***p < 0.001, ****p < 0.0001

To further validate the bone repairability of our hybrid hydrogels from micro aspects, histological examinations were performed by HE staining and MT staining. HE staining demonstrated that the margin of the defect treated with the F-G/B-M hydrogel was covered by newly formed bone. The sham and G groups showed only little bone tissue and massive fibrous tissue connected with the defects (Fig. 7). As further illustrated in Additional file 1: Fig. S6, the percentage of regions of newly formed osseous tissue was significantly higher in the F-G/B-M group (86.4 ± 3.45% at 4 weeks and 90.3 ± 3.94% at 8 weeks) than in the G/M group (59.9 ± 5.43% at 4 weeks and 63.0 ± 5.33% at 8 weeks) and sham group (5.3 ± 2.28% at 4 weeks and 9.6 ± 3.22% at 8 weeks). Due to the deposition of a large amount of collagen matrix, the early formed bone was reconstructed as normal lamellar bone, stained red in Masson staining [36]. Masson trichrome staining displayed the formation of an osteoid matrix with increased newly formed blood vessels at the edge of the defect in the F-G/B-M group. In contrast, defects treated with the sham and G hydrogels were filled with fibrous tissue with little new bone (Additional file 1: Fig. S5). Notably, both at 4 weeks and 8 weeks, the group with BMP-2 (G/B-M and F-G/B-M) showed significantly thicker bone trabeculae than other groups, while the group with bFGF (F-G/M and F-G/B-M) featured more neovascularization. We also performed immunofluorescence staining to assess both neovascularization and osteogenesis. For the analysis of the degree of ossification, we first assessed the osteogenic marker OCN and Col-I expression. The representative images are presented in Fig. 8, and consistent results also revealed that the most and strongest positive staining was observed in the F-G/B-M group. CD31 is expressed by endothelial cells to mediate intercellular adhesion and involve in the formation of inter-endothelial junctions [37]. Therefore, it is widely used for assessing revascularization in vivo. As shown in Additional file 1: Fig. 9A, the neovasculature was stained red and circular, clustered predominantly in the surrounding connective tissue, which was partially co-localized with the neogenic bone tissues. At 4 weeks, bone endothelial cells in the groups with bFGF (F-G/M and F-G/B-M) were strongly positive for CD31. Comparatively, CD31 expression was rarely observed in other groups. At 8 weeks, more CD31 immunofluorescence localization in the new blood vessels was found in the F-G/B-M group (Fig. 9B), while a limited number of the vascular lumen with smaller diameter was identified in other groups. Moreover, it was observed that although compared to the F-G/M group, more bone matrix formation was observed in the G/B-M group, as well as expression of osteogenic markers (Figs. 7, 8), the vascularization in the G/B-M group was delayed and not present until the eighth week (Additional file 1: Fig. S5). Thus, the F/G-B/M group demonstrated the optimal vascularized bone regeneration performance, which might be attributed to the coupling of osteogenesis and angiogenesis.

## Discussion

Despite the biological complexity involved in the bone healing process, most prevalent therapeutic approaches focused only on one or two aspects of this highly delicate situation. Inspired by spatiotemporal presentation cascade of morphogens during natural bone healing, we developed a hybrid hydrogel-based delivery system harnessing the macromolecule delivery feature of both GelMA hydrogel and mineral coating to optimally orchestrate the coupling of osteogenesis and angiogenesis for the vascularized bone regeneration. Taking advantage of the two materials components used in this study, we successfully achieved dual growth factor delivery with distinctive release profiles: bFGF was released in a "burst" pattern to promote early-stage angiogenesis, while a more durable delivery of BMP-2 facilitated osteogenesis.

In vitro experiments confirmed that the hybrid hydrogel facilitated the proliferation and adhesion of HUVECs and BMSCs and promoted ALP expression and calcium nodule deposition, together with upregulation of osteogenic and angiogenic gene expression. Subsequent in vivo experiments indicated that this hybrid hydrogel also presented enhanced and coupled osteogenesis and angiogenesis in a critical-size defect model. Thus, such design of our F-G/B-M hydrogel resembles more closely the natural bone regeneration process, which is one step forward to the clinical application of these strategies [38].

Bone healing is a multi-stage process involving four dynamic temporal phases that overlap each other: inflammation phase, soft callus formation phase, hard callus formation phase, and bone remodelling phase [39, 40]. During the early phages, it is crucial that vascular ingrowth into the hematoma to form a soft callus in which bFGF is involved, while during the late stages, the onset of hard callus formation and bone remodelling is continuously induced by BMP-2 [40]. The hybrid hydrogel we developed met this requirement, simulating the release profile of growth factors at different phases. Specifically, to mimic the release of bFGF during the early stage, bFGF encapsulated in GelMA was released in a "burst" release manner, attributed to the hydrogel's high permeability network. In contrast, to mimic the release of BMP-2 during hard callus formation and bone remoulding phase, sustained release of BMP-2 was achieved mainly owing to the continuous degradation of the mineral coating on MCMs. The appropriate release period for BMP-2 in bone regeneration has been reported to be 2 weeks to 4 weeks [41]. In our designed hydrogel, MCMs precisely played an important role in the controlled release of BMP-2. The sustained release of BMP-2 was synchronous with time and approached its plateaus at 4 weeks (Fig. 3). In addition, although a variety of delivery systems employing polymers as building blocks have been developed [42–44], the MCMs in GelMA offered improved osteoinductivity and the ability to mimic the natural bone microenvironment.

To date, some studies have evidenced the bone regenerative effect of dual GFs released in a sequential and controlled manner [2, 45]. Nevertheless, while the majority of dual growth factors delivery systems chose BMP-2, only a few focused on the co-delivery of bFGF. In contrast to other common angiogenic growth factors such as VEGF, bFGF offers more diverse capabilities. Firstly, bFGF regulates angiogenesis in various steps, from basement membrane degradation and integrin expression to endothelial cell proliferation and migration, vascular maturation and basement membrane remodelling [46]. Secondly, bFGF also stimulates the proliferation of BMSCs [47]. Montero et al. reported that disruption of the bFGF gene led to reduced bone mass as well as decreased bone formation in mice [48]. Lastly, it was found that bFGF is essential for the role of BMP-2 in bone anabolism. bFGF potentially stimulates the proliferation of local osteoprogenitor cells, whereas BMPs subsequently enable the development of the enlarged osteoprogenitor cell pool toward the osteoblast lineage [49, 50]. In summary, bFGF not only serves as a potent inducer of angiogenesis but also exerts a positive effect on the regulation of osteogenesis-related cells and growth factors.

We found that the dual GFs group displayed the highest expression of osteogenic marker RUNX2 and osteogenic-related genes (Fig. 4) from in vitro experiments. Similarly, in vivo studies also revealed that the F-G/B-M group had the greatest amount of bone matrix formation (Figs. 6, 7). To interpret the above results, our further studies demonstrated that the dual GFs group had the most amount of vascular formation as well as the mature bone matrix appeared in the perivascular area, which elucidated the significance of bFGF-induced angiogenesis for bone regeneration. As a key regulator of angiogenesis, bFGF involves several processes, including degradation and reformation of basement membrane and proliferation and migration of endothelial cells [51]. Given its superiority in angiogenesis, bFGF has been used extensively for tissue engineering. For instance, Fan et al. have developed a kind of glutathione-modified collagen hydrogel loaded with bFGF to promote angiogenesis in the infarction region [52]. In the present study, bFGF loaded in GelMA exhibited a "burst" release in the early stage, promoting intracellular nitric oxide expression and upregulation of a range of angiogenic-related genes, consequently stimulating vascularization. Meanwhile, bFGF, together with BMP-2, regulates the activity of osteoblast-associated cells, which eventually facilitated the coupling of angiogenesis and osteogenesis. In addition, as shown in Fig. 9, more CD31-positive endothelial cells could be found in the G/M group compared to the G group. Certain studies reported that Ca^2+^ may stimulate the secretion of vascular-associated cytokines and contribute to the adhesion and proliferation of endothelial cells, as well as capillary formation [53], so we assumed that the Ca^2+^ released from the MCM affected the angiogenesis.

## Conclusion

In the current research, we successfully developed a dual growth factor delivery system that can mimic the natural bone repair process of autologous bone. This hybrid hydrogel was designed to enable differentiated delivery modes of BMP-2 and bFGF, which could orchestrate osteogenesis and angiogenesis. The original components (GelMA, MCM, and GFs) of the material confer appropriate physical and biological characteristics to the system, while its degradation products (calcium and phosphate) participate in the long-term regulation of physiological activities as well. In vitro studies demonstrated that this hybrid hydrogel could potently enhance the osteogenic differentiation of hBMSCs and capillary formation of HUVECs. Furthermore, in-depth in vivo studies illustrated the capability of the designed hydrogel to induce vascularized bone regeneration. Notably, our hybrid hydrogels are obtained from naturally derived materials with defined biological activity, which comes closer to safe and effective bone repair materials. This work may provide a novel perspective on the design of bionic delivery systems and spark widespread interest in the field of bone tissue engineering based on growth factors.

## Fundings

This work was supported by National Key Research and Development Projects (2018YFC1105400), National Natural Science Foundation of China (81872173, 82072959, 31870959).

## Supplementary Information

**Additional file 1: Table S1.** Primer pairs used in the qRT-PCR studies. **Fig. S1.** Characterizations **Fig. S2.** Biocompatibility evaluation. **Fig. S3.** In vitro osteogenesis potential. **Fig. S4.** Effect on intracellular NO expression of HUVECs, **Fig. S5.** Masson's trichrome staining. **Fig. S6.** The quantitative results of H&E staining.

## Data Availability

The data used to support the findings of this study are available from the corresponding author upon reasonable request.
