# Supplementary material for: Spatiotemporal regulation of angiogenesis/osteogenesis emulating natural bone healing cascade for vascularized bone formation
Source: J Nanobiotechnology. 2021 Dec 14;19:420. doi: 10.1186/s12951-021-01173-z (PMC8670285; doi:10.1186/s12951-021-01173-z)
Supplement: Supplementary file 1 — Additional file 1: Table S1. Primer pairs used in the qRT-PCR studies. Fig. S1. Characterizations Fig. S2. Biocompatibility evaluation. Fig. S3. In vitro osteogenesis potential. Fig. S4. Effect on intracellular NO expression of HUVECs, Fig. S5. Masson's trichrome staining. Fig. S6. The quantitative results of H&E staining. [file 12951_2021_1173_MOESM1_ESM.docx]

**Additional Material**

**Table S1.** Primer pairs used in the qRT-PCR studies

| **Primer name** | **Primer sequence (5’-3’)** |
| --- | --- |
| **ALP(F)** | ACCACCACGAGAGTGAACCA |
| **ALP(R)** | CGTTGTCTGAGTACCAGTCCC |
| **Runx2(F)** | CTTGACCATAACCGTCTTCA |
| **Runx2(R)** | GTCATCAATCTTCTGTCTGT |
| **Col I(F)** | GTGCGATGACGTGATCTGTGA |
| **Col I(R)** | CGGTGGTTTCTTGGTCGGT |
| **OCN(F)** | GGCGCTACCTGTATCAATGG |
| **OCN(R)** | GTGGTCAGCCAACTCGTCA |
| **Osterix(F)** | CCTCTGCGGGACTCAACAAC |
| **Osterix(R)** | AGCCCATTAGTGCTTGTAAAGG |
| **CD31(F)** | CCAAGGTGGGATCGTGAGG |
| **CD31(R)** | TCGGAAGGATAAAACGCGGTC |
| **VEGF-A(F)** | AGGGCAGAATCATCACGAAGT |
| **VEGF-A(R)** | GGGTCTCGATTGGATGGCA |
| **ANG(F)** | CTGGGCGTTTTGTTGTTGGTC |
| **ANG(R)** | GGTTTGGCATCATAGTGCTGG |
| **vWF(F)** | CCTTGACCTCGGACCCTTATG |
| **vWF(R)** | GATGCCCGTTCACACCACT |
| **β-actin(F)** | CCTCGCCTTTGCCGATCC |
| **β-actin(R)** | GGATCTTCATGAGGTAGTCAGTC |

**
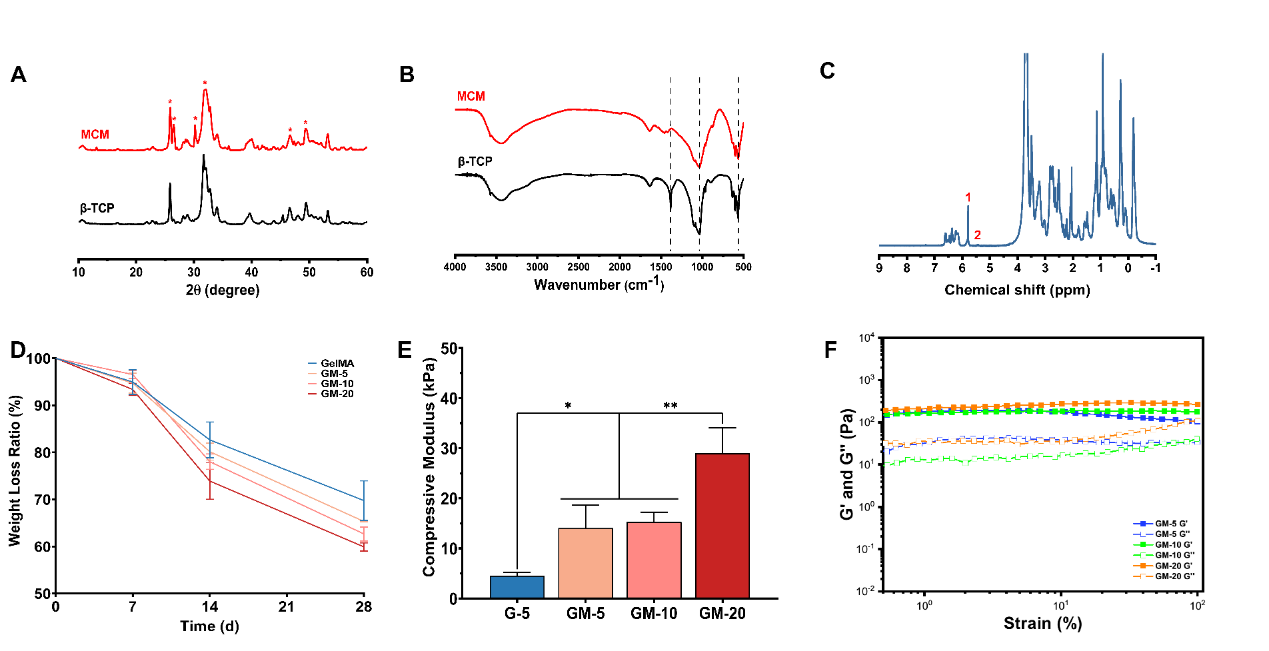
**

**Fig. S1.** Characterizations. **(A)** Small-angle X-ray scattering and **(B)** FTIR spectra of β-TCP and MCM. **(C)** ¹H NMR spectra of GelMA. **(D)** Weight loss analysis and **(E)** Mechanical test of GelMA/MCM. Statistically significant differences are indicated with *p < 0.05, **p < 0.01.

**
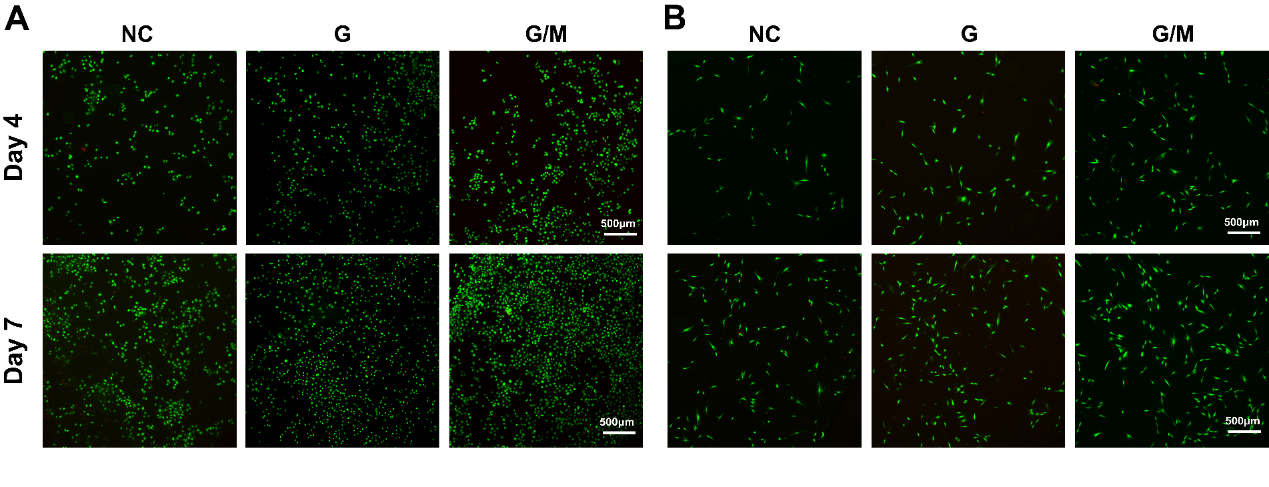
**

**Fig. S2.** Biocompatibility evaluation. Representative Live/Dead images of **(A)** HUVECs and **(B)** BMSCs at day 4 and 7.

**
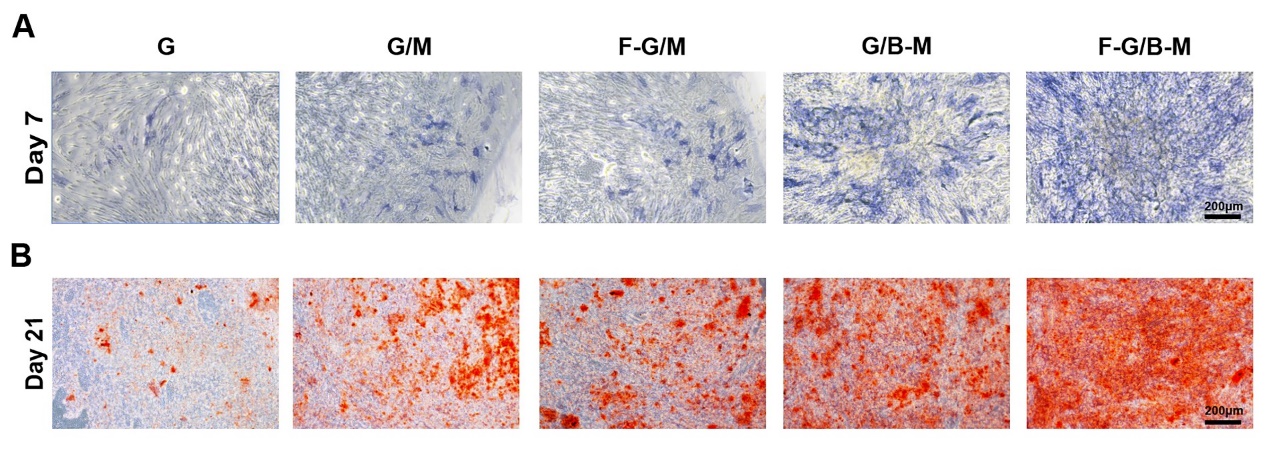
**

**Fig. S3.** In vitro osteogenesis potential. **(A)** Representative ALP staining images on day 7. **(B)** Representative ARS images on day 21.

**
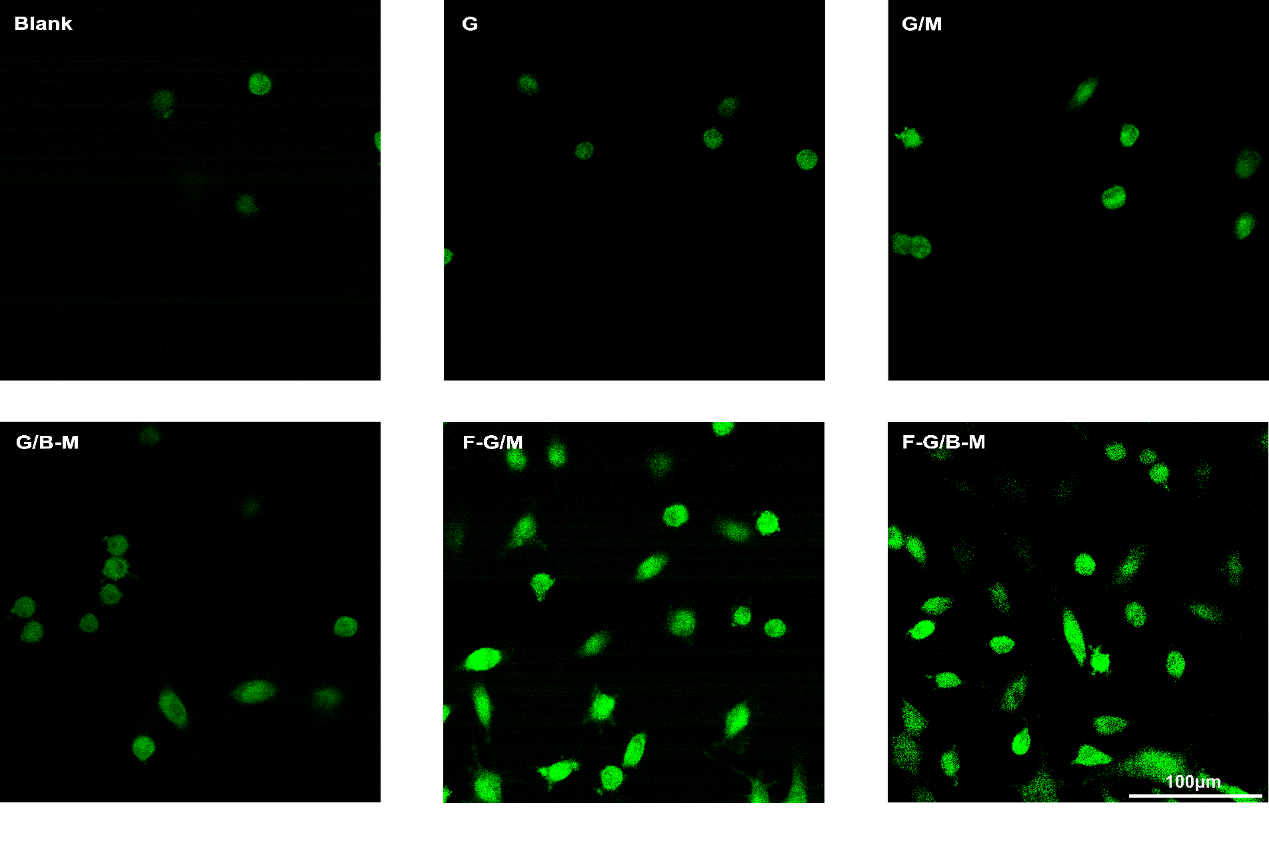
**

**Fig. S4.** Effect on intracellular NO expression of HUVECs.

**
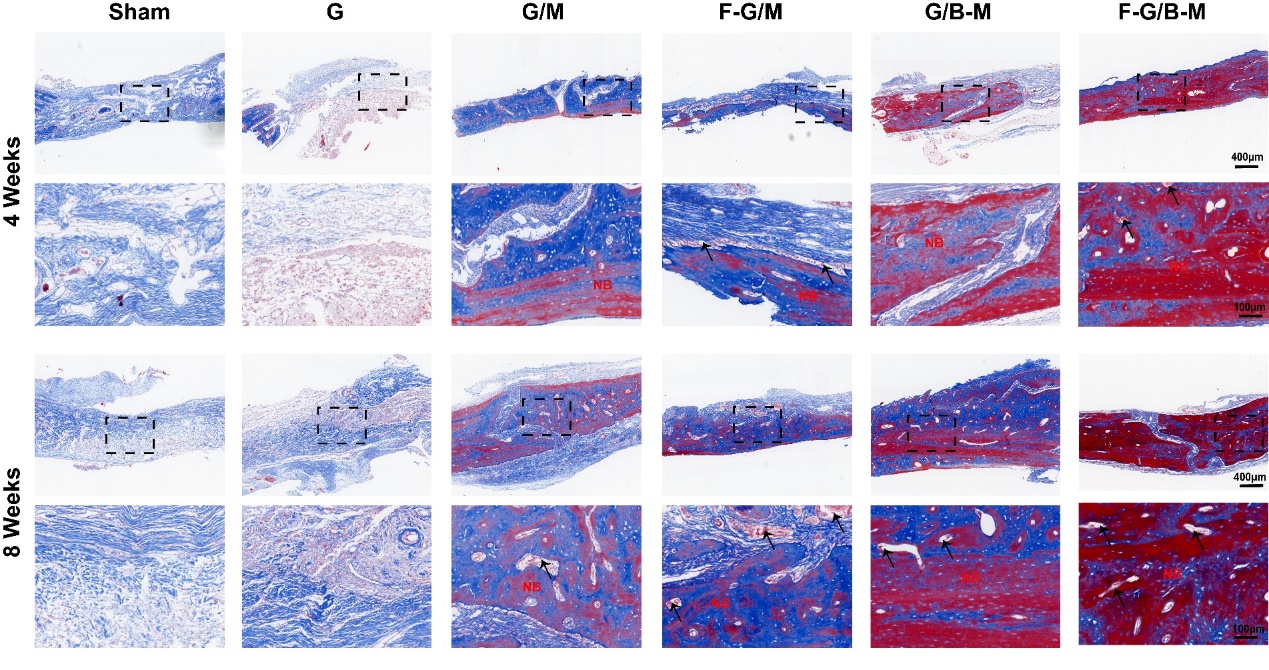
**

**Fig. S5.** Masson's trichrome staining of newly formed bone in the defect after implantation with different hydrogels for 4 and 8 weeks. Representative images were observed by low magnification and high magnification, showing the newly mineralized bone tissue (NB) and vascular formation (black arrow).


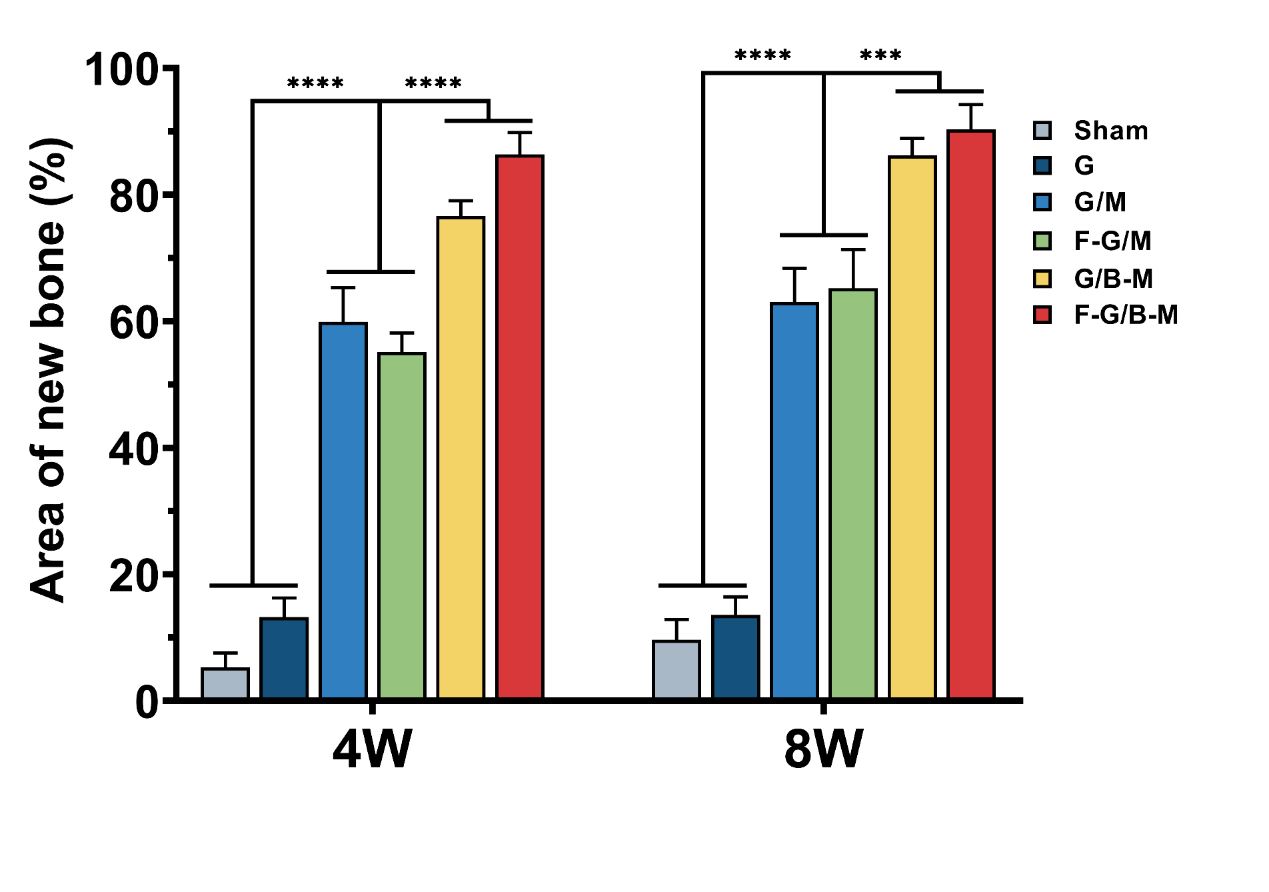


**Fig. S6.** Hematoxylin and Eosin (H&E) staining of newly formed bone in the defect after implantation with different hydrogels for 4 and 8 weeks. The quantitative results of the newly formed bone area after 4 weeks and 8 weeks. Statistically significant differences are indicated with *p < 0.05, **p < 0.01, ***p < 0.001, ****p < 0.0001.
